# Commensal gut microbiota-derived acetate and propionate enhance heart adaptation in response to cardiac pressure overload in mice

**DOI:** 10.7150/thno.76002

**Published:** 2022-10-17

**Authors:** Chen-Ju Lin, Yu-Che Cheng, Hung-Chih Chen, Yu-Kai Chao, Martin W. Nicholson, Eric C.L. Yen, Timothy J. Kamp, Patrick C.H. Hsieh

**Affiliations:** 1 Institute of Biomedical Sciences, Academia Sinica, Taipei 115, Taiwan.; 2 Department of Nutritional Science, University of Wisconsin-Madison, Madison, WI 53705, USA.; 3 Department of Medicine and Stem Cell and Regenerative Medicine Center, School of Medicine and Public Health, University of Wisconsin-Madison, Madison, WI 53705, USA.; 4 Institute of Medical Genomics and Proteomics and Institute of Clinical Medicine, National Taiwan University, Taipei 106, Taiwan.

**Keywords:** Gastrointestinal Microbiome, Cardiac Hypertrophy, Acetate, Propionate, Myofibroblasts

## Abstract

**Background:** The gut microbiota plays a vital role in maintaining tissue homeostasis and regulating disease pathophysiology; however, the underlying mechanisms remain to be elucidated. We previously showed that mice depleted of gut microbiota with antibiotics (ABX mice) were more prone to cardiac rupture after infarction, suggesting that the gut microbiota impacts cardiac structural remodeling following injury. Here, we aimed to determine whether the gut microbiota is required for adaptive cardiac remodeling in response to pressure overload stress.

**Methods:** Transverse aortic constriction (TAC) surgery was performed and cardiac function was evaluated by echocardiography and catheterization, followed by mechanical tests and extracellular matrix (ECM) studies. Germ-free mice with cecal microbiota transplantation were used for validation. 16S ribosomal DNA sequencing and PICRUSt2 analysis were applied to predict the key metabolic pathways. ABX mice were supplemented with the derived metabolic products and their efficacy was tested. To elucidate the underlying mechanism, we isolated mouse primary cardiac fibroblasts and treated them with the metabolites. Lastly, G-coupled protein receptor 41 (GPR41) and GPR43 double knockdown cardiac fibroblasts were generated and the anti-fibrogenic effect of metabolites was determined.

**Results:** Cardiac hypertrophy and dysfunction were more severe in ABX-TAC mice compared to the controls. Moreover, TAC-induced fibrosis was more profound in ABX hearts, which was accompanied by disrupted ECM structure making the heart tissues mechanically weaker and more brittle. Reconstruction of healthy gut microbiota in germ-free mice successfully restored cardiac function and prevented excessive fibrosis and ECM disarray under stress. Furthermore, functional prediction identified acetate and propionate as critical mediators in the gut microbiota-modulated cardiac mechanics. Supplementation of acetate and propionate improved heart function, attenuated fibrosis, and reversed ECM disarray after TAC. In addition, treating primary cardiac fibroblasts with acetate and propionate attenuated cell contraction, inhibited myofibroblast formation, and reduced collagen formation after TGF-β1 stimulus. Finally, knocking down GPR41 and GPR43 receptors in cardiac fibroblasts blunted the inhibitory effects of acetate and propionate.

**Conclusions:** The gut microbiota is a potential therapeutic target for cardiac ECM remodeling and heart structural integrity. By establishing a healthy gut microbiome or replenishing the derived metabolites, we could improve cardiac health under dysbiosis after pressure-overload stress.

## Introduction

The gut microbiota has been implicated in the pathogenesis and prevention of cardiovascular diseases, which remain a leading cause of death worldwide 1, through manipulation of host immune and metabolic homeostasis 2, 3. It has been shown to play both beneficial and adverse roles in animal disease models and patients, which could be attributed to the heterogeneity of the microbial community. In our previous studies, we reported that mice deprived of gut microbiota were susceptible to cardiac rupture after myocardial infarction 4, implying that the gut microbiota plays a role in the maintenance of heart structural integrity. This gave rise to the hypothesis that the gut microbiota and metabolites may impact heart biomechanics.

Modifications in composition and biomechanical properties of the extracellular environment are required for normal heart development as well as heart remodeling during disease progression 5. Particularly, changes in stiffness, dimensionality, and geometry of the extracellular matrix (ECM) were able to alter cell behavior and phenotype, which ultimately altered organ function 6. For example, constant changes in cardiac stiffness via modification of total collagen amount and the ratio of collagen I to collagen III in response to mechanical stress are essential during heart development 7. In addition to physiological homeostasis, pathological remodeling in terms of ECM structure, composition, and porosity were found in failing hearts 8. Serving as the scaffold supporting heart structure, ECM remodeling after cardiac injury prevents hearts from rupturing. This is mainly attributed to the activation of cardiac fibroblasts 9, which induce collagen synthesis that enhances the strength of the heart to compensate for post-injury cardiomyocyte loss. However, in the long term, excessive collagen accumulation leads to ventricular stiffening that eventually diminishes both the diastolic and systolic functions of the heart.

Excessive ECM deposition, a pathological process causing dysfunction and failure in multiple organs, has been linked directly to the gut microbiota. For instance, intestinal fibrosis was found to be aggravated in the presence of bacterial lipopolysaccharide via interacting with toll-like receptor 4 that suppresses SMAD7 expression and promotes transforming growth factor beta 1 (TGF-β1) signaling and collagen formation 10. In a mouse model of liver fibrosis, lipopolysaccharide activates hepatic stellate cells via toll-like receptor 4 and enhances TGF-β1 signaling through the Bambi-yD88-NF-kB pathway 11. Other than direct interaction, gut microbiota could also produce metabolites regulating fibrogenesis. Trimethylamine N-oxide, a coproduct of gut microbiota and host metabolism, has been shown to promote renal fibrosis in association with SMAD3 activation 12. Through systemic circulation, trimethylamine N-oxide has also been found to promote TGF-β1 signaling in cardiac fibroblasts by upregulating TGF-β receptor 1 expression and enhancing SMAD2 activation 13.

Given the heterogeneity and complexity of the human microbiome, the therapeutic potential of the gut microbiota and its metabolites is undetermined. In this study, we sought to unveil the role of gut microbiota in cardiac ECM homeostasis and remodeling in response to pressure overload.

## Methods

### Animals

All experiments involving animals were conducted according to the U.S. National Institutes of Health Guide for the Care and Use of Laboratory Animals. All animal protocols were approved by the Institutional Animal Care and Use Committee of Academia Sinica. The animal protocol for germ-free mouse experiments was approved by the Institutional Animal Care and Use Committee of the National Laboratory Animal Center, Taiwan.

C57BL/6J mice were purchased from the National Laboratory Animal Center, Taiwan. Eight- to ten-week-old animals were used for the experiments conducted in this study. Mice under different treatments were housed separately in individually ventilated cages in the Academia Sinica Specific Pathogen-Free Animal Facility. Eight-week-old germ-free male C57BL/6J mice were maintained in plastic flexible film gnotobiotic isolators under a strict 12-h light cycle and fed autoclaved water and diet in the Germ-Free Mouse Facility at the National Laboratory Animal Center, Taiwan. The mice were numbered and the cages were housed in random order on the shelves. All measurements were conducted in a random order, and the investigators were blinded to the treatment groups. The number of mice used in each experiment is indicated by the dot number in each figure.

### Surgery and heart function assessment

To induce pressure-overload stress, loose knots were tied around the transverse aorta and a small piece of the 27½ gauge blunt needle was placed parallel to the transverse aorta. The first knot was quickly tied against the needle, followed by the second, and the needle was swiftly removed, leaving a 0.4 mm-diameter in the constricted site of the aorta. Three days after surgery, the constricted site of the aorta was identified by color Doppler imaging, and the peak aortic velocity at the constricted site was derived from pulse wave Doppler echocardiography. To induce cardiac hypertrophy in germ-free mice, osmotic pumps (1004, ALZET, DURECT) filled with Angiotensin-II (Sigma) were subcutaneously implanted into the mice with a continuous dose of 1.44 μg/g/day for 28 days. Twenty-eight days after surgery, cardiac function was assessed by echocardiography using Vivid-q Ultrasound equipped with a 5-13 MHz intraoperative probe (GE). Mice were subjected to cardiac catheterization the next day. Mice were anesthetized with 1 g/kg urethane intraperitoneally around 15 min before catheter insertion with supplemental injections of 0.144 g/kg each time until the mice reached the surgical depth of anesthesia. The mice were in a supine position and kept on a heating pad to maintain body temperature at 37 °C and supported by a ventilator with 100% O_2_ throughout the surgery. A pressure-volume micro-catheter (1.4F PVR-839 catheter, Millar) was inserted into the left ventricle through the right carotid artery of the mice. Intra-cardiac pressure (P) and volume (V) were measured and real-time data were acquired by a P-V conductance system (MPVS Ultra, AD Instruments) coupled to a digital converter (PowerLab, ADInstruments). To measure heart function, a graded preload reduction was induced by compressing the inferior vena cava through the diaphragm. End-systolic pressure-volume relationship (ESPVR) was obtained and used as an indicator of systolic function and end-diastolic pressure-volume relationship (EDPVR) as that of diastolic function. Data were analyzed using physiological data analysis software (LabChart Pro, ADInstruments).

### Antibiotic treatment

A broad-spectrum antibiotic cocktail for induction of dysbiosis in SPF mice was prepared by dissolving ampicillin (0.25 g/L), metronidazole (0.25 g/L), neomycin (0.25 g/L), and vancomycin (0.125 g/L) (all products of Sigma) in autoclaved water. For dose-dependency tests, one half and one eighth of the dose were used. To induce dysbiosis in SPF mice, the antibiotic cocktail was administered seven days before surgery or seven days before fecal microbiota transplantation.

### Acetate and propionate supplement

To prepare the acetate and propionate supplement, acetate (200 mM), propionate (200 mM), or a mixture (100 mM acetate and 100 mM propionate) (both products of Sigma) were dissolved in autoclaved water. pH and sodium-matched autoclaved water was given as a control treatment. To test the efficacy of acetate and propionate in mice with dysbiosis, the described solution was given along with antibiotics (concentrations as listed in the “Antibiotic Treatment” section) seven days before surgery.

### Fecal microbiota transplantation

For the preparation of fecal microbiota transplantation materials, cecal contents from control mice with sham or TAC surgery were resuspended in phosphate-buffered saline (PBS) (50 μg cecal contents/1 mL of PBS). For SPF mice, fecal microbiota transplantation was conducted by oral gavage starting one day after ABX treatment, followed by two more doses given every other day with a last dose given two days before surgery. For germ-free mice, three doses of fecal microbiota transplantation were given every other day until two days before surgery. Each dose contained 300 μL of the fecal microbiota transplantation materials.

### 16S ribosomal DNA sequencing and PICRUSt2 analysis for microbial functional prediction

Bacterial genomic DNA was extracted from approximately 200 mg of cecal contents using the innuSPEED stool DNA kit (Qiagen) according to the manufacturer's instructions. The full-length 16S genes were amplified by PCR with barcoded primers (forward: 5'- Phos/GCATC- 16-base barcode -AGRGTTYGATYMTGGCTCAG -3' and reverse: 5'-Phos/GCATC- 16-base barcode - RGYTACCTTGTTACGACTT -3', degenerate base R = A, G; Y = C, T; M = A, C) mixed with KAPA HiFi HotStart ReadyMix (Roche). The PCR products were examined by electrophoresis on 1% agarose gel and those with the main strip ~1500 bp were then purified by the AMPure PB Beads (PacBio) for SMRTbell library (PacBio) preparation. Pooled amplicon libraries were sequenced in the circular consensus sequence mode on a PacBio Sequel IIe instrument to generate the HiFi reads with a Phred quality score of 30. The circular consensus sequence reads were obtained through the SMRT Link software (PacBio) followed by DADA2 v.1.10.1 processing to obtain amplicons with single-nucleotide resolution from the full-length 16S rDNA genes 14. Each amplicon sequence was then annotated to taxonomy classification based on the NCBI database (2020.7) using QIIME2 v.2020.11.1 15. Alpha diversity of the microbial community including the number of observed species, Shannon index, and Simpson index was analyzed using QIIME2. Beta diversity was determined using the phyloseq and Rtsne packages in R v.3.3.1. For functional prediction analysis, 16S rDNA sequencing data were analyzed using PICRUSt2 and MetaCyc pathway abundances were calculated 16.

### Statistics

Statistical analysis was performed by GraphPad Prism. Results are presented as mean ± SEM. The Kaplan-Meier method and log-rank (Mantel-Cox) test were used to construct and compare the survival curves, respectively. One-way ANOVA with the Tukey adjustment for multiple comparisons was used to compare the effects of FMT, acetate, and propionate treatments. Two-way ANOVA with the Tukey adjustment for multiple comparisons was used to compare the effects of ABX treatment and TAC surgery. An unpaired two-tailed t-test was used to compare the difference in microbial alpha diversity and composition between healthy and diseased FMT donors and recipients. A P value of less than 0.05 was considered statistically significant.

Other detailed methods are available in the Data Supplement.

## Results

### Depletion of gut microbiota exacerbates heart dysfunction and dilation

**Figure 1:**
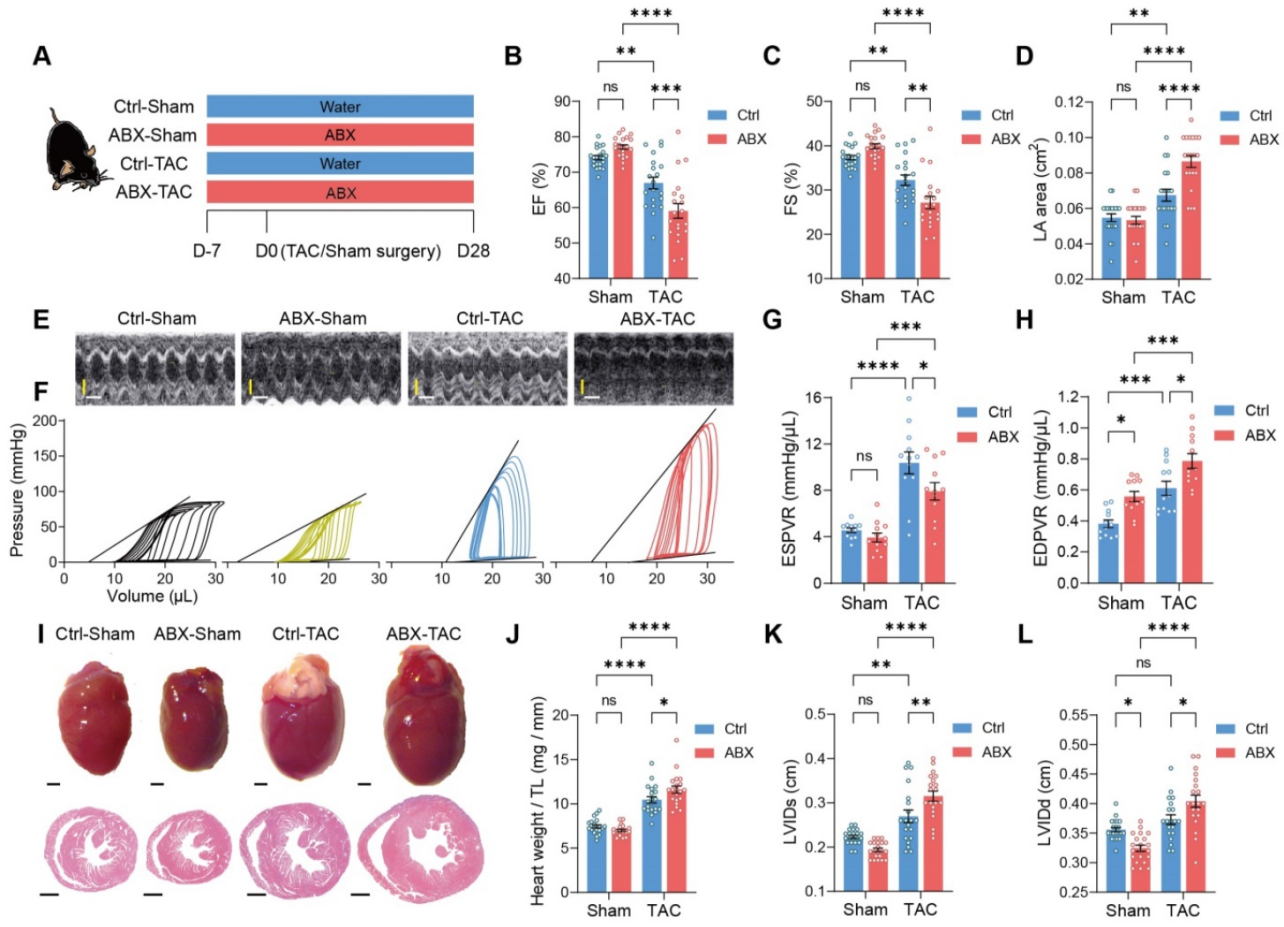
** Depletion of gut microbiota impairs heart function and exacerbates left ventricular dilatation after pressure overload. A**, Experimental design for testing the effect of gut microbiota depletion on mice after transverse aortic constriction (TAC). **B** through **E**, Heart systolic function was estimated by ejection fraction (EF) (**B**) and fractional shortening (FS) (**C**); diastolic function was estimated by left atrial (LA) area (**D**) via echocardiography as shown in representative M-mode images (**E**). Scale bar, 0.1 s (white horizontal bar) and 1 mm (yellow vertical bar). **F** through **H**, Heart function was assessed by cardiac catheterization-derived pressure-volume loops (**F**); end-systolic pressure-volume relationship (ESPVR) slope representing cardiac contractility serves as systolic function indicator (**G**) whereas end-diastolic pressure-volume relationship (EDPVR) slope measuring myocardial passive stiffness is a diastolic function indicator (**H**). **I**, Cardiac hypertrophy and enlargement of left ventricular internal dimensions were shown by representative bright-field images of whole hearts and hematoxylin and eosin stained heart sections. Scale bar, 1 mm.** J**, Cardiac hypertrophy was estimated by heart weight normalized by tibial length (TL). **K** and **L**, left ventricular dilation was measured by left ventricular internal diameter at end-systole (LVIDs) (**K**) and LVID at end-diastole (LVIDd) (**L**). Statistical significance was determined by two-way ANOVA with Tukey multiple comparisons test. ns, not significant; *, *P* < 0.05; **, *P* < 0.01; ***, *P* < 0.001; ****, *P* < 0.0001.

To test the hypothesis that the gut microbiota modulates cardiac remodeling after pressure overload stress, we depleted the gut microbiota from mice by delivering antibiotics (ABX) in the drinking water seven days before transverse aortic constriction (TAC) surgery **(Figure 1A)**. The gut microbiota-depletion efficiency of ABX was determined after seven days of treatment (**Figure S1A**). We found that ABX significantly reduced gut microbiota in a dose-dependent manner. The heart function of the mice was evaluated 28 days after surgery. TAC surgery induced systolic dysfunction in both Ctrl and ABX mice but a more severe systolic dysfunction was found in ABX-TAC mice compared to Ctrl-TAC mice, as evidenced by significantly lower ejection fraction (EF) and fractional shortening (FS) (**Figure 1B** and **1C**). Moreover, TAC surgery induced left atrial enlargement in Ctrl mice but induced a greater elevation in ABX mice, suggesting that ABX-TAC mice had exacerbated diastolic dysfunction (**Figure 1D**). Both echocardiography and cardiac catheter-derived pressure-volume (PV) plots showed exacerbated heart dysfunction in ABX-TAC mice **(Figure 1E** and **1F)**. The PV plot shows raised pressure in both control-TAC and ABX-TAC mice compared to their sham counterparts, confirming that TAC surgery-induced pressure overload in both groups of animals. This is also demonstrated by the elevation of resting pressure at end-systole and end-diastole in both control-TAC and ABX-TAC mice compared to their sham counterparts (**Figure S2**). Interestingly, ABX-TAC mice had a significantly higher left ventricular pressure at end-diastole than control-TAC mice. In addition, TAC induced a decrease in the slope of PV relationship at end-systole (ESPVR), which was more profound in ABX mice, indicating a greater reduction in cardiac contractility **(Figure 1G)**. Furthermore, an increase in the slope of PVR at end-diastole (EDPVR) after TAC surgery was aggravated in ABX mice, implying intensified left ventricular (LV) stiffening **(Figure 1H)**. Notably, on day 0 (day of surgery) **(Figure S1A)**, there was no difference in echocardiography-derived parameters, including EF and FS **(Figure S1B** and **S1C)**. Although a drop of around 17% in the early diastolic mitral annular velocity (e') was found in ABX mice **(Figure S1D)**, the ratio of the peak early mitral inflow velocity over e' (E/e') was unchanged **(Figure S1E)**. However, hemodynamic parameters- end-diastolic volume was dose-dependently decreased by ABX treatment and end-systolic pressure tended to be decreased in ABX mice (**Figure S1F**-**S1H**). Interestingly, although there was a change in the ESPVR slope, the EDPVR slope was elevated by ABX treatment dose-dependently **(Figure S1I** and **S1J)**, implying that ABX was able to impact cardiac diastolic function with LV stiffening. Next, to estimate the level of cardiac hypertrophy, we dissected the hearts following sacrifice at day 28 and captured the freshly isolated hearts for histological studies. We found that TAC-induced cardiac hypertrophy was more severe in ABX mice **(Figure 1I)**. This was supported by greater heart weight and larger LV internal diameters **(Figure 1J**-**1L)**. Surprisingly, ABX treatment could readily change heart morphology and LV volume **(Figure S1K)**. This is shown by a dose-dependent decrease in heart weight, end-systolic volume (ESV), and end-diastolic volume (EDV) **(Figure S1L-S1N)**. These results indicate that pre-depletion of gut microbiota using ABX treatment worsens cardiac outcomes after TAC surgery, leading to worse systolic and diastolic function, as well as exacerbated LV dilation and cardiac hypertrophy.

### Heart mechanics and ECM remodeling are disturbed in gut microbiota-depleted mice

**Figure 2:**
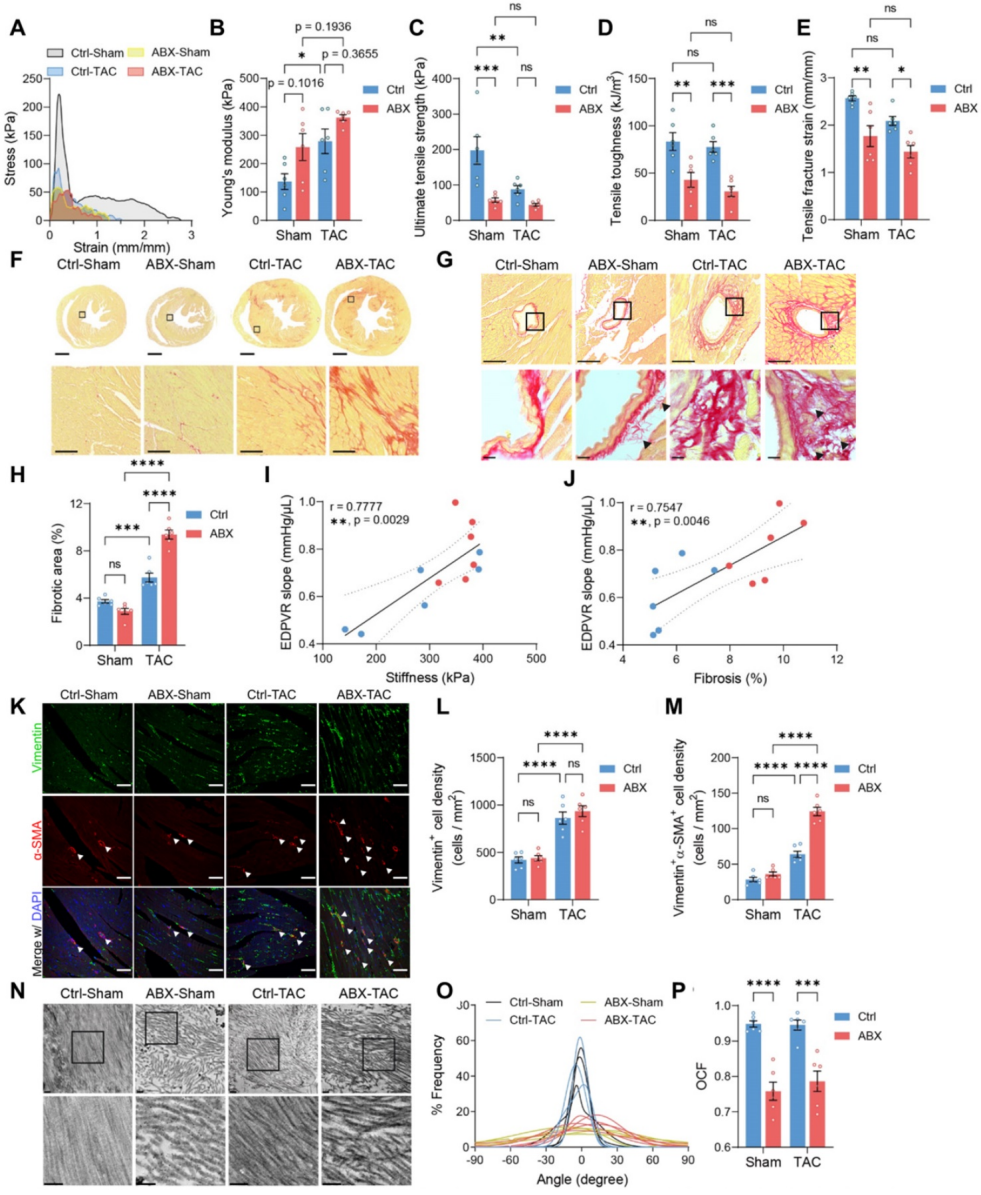
** Mechanical strength and cardiac ECM ultrastructure are disturbed by depletion of gut microbiota under stress. A**, Representative stress-strain curves derived from the tensile test on mouse hearts. **B** through **E**, Tensile test-derived mechanical properties including Young's modulus (**B**), Ultimate strength (**C**), tensile toughness (**D**), and fracture strain (**E**) of mouse hearts. **F** and **G**, Representative picrosirius red-stained heart sections showing global, interstitial fibrosis; upper scale bar, 1 mm; lower scale bar, 100 µm (**F**), and perivascular fibrosis; upper scale bar, 100 µm; lower scale bar, 10 µm (**G**). **H**, Quantification of fibrotic area. **I**, Correlation analysis of end-diastolic pressure-volume relationship (EDPVR) slope and heart tissue stiffness. Simple linear regression was performed. The solid line indicates the best-fit line, the dashed lines above and below it indicates the upper and lower limit of the 95% confidence intervals of the linear regressions, respectively, and r is the correlation coefficient. **J**, Correlation analysis of EDPVR slope and fibrosis. **K**, Immunofluorescent staining with vimentin and alpha-smooth muscle actin (α-SMA). Scale bar, 50 µm. **L** and **M**, Quantification of vimentin-positive cells representing cardiac fibroblasts (**L**) and vimentin and α-SMA double-positive cells representing myofibroblasts (**M**). **N**, Collagen fibrils under the transmission electron microscope. Scale bar, 0.2 µm. **O**, Collagen fibril angular distribution. **P**, Orientation correlation function (OCF) for quantification of fiber alignment. Statistical significance was determined by two-way ANOVA with Tukey multiple comparisons test in **A** through **E**, **H**, **L**, **M**, and **P**. ns, not significant; *, *P* < 0.05; **, *P* < 0.01; ***, *P* < 0.001; ****, *P* < 0.0001.

To investigate if worsened heart function and hypertrophy in ABX mice after TAC were caused by the change in heart mechanics, we did tensile and compressive tests to estimate the mechanical properties of the hearts. Stress-strain curves obtained from the tensile test were used to derive the following mechanical indices **(Figure 2A)**. Young's modulus tends to increase in ABX hearts, indicating that ABX treatment leads to heart stiffening **(Figure 2B)**. Moreover, both TAC surgery and ABX treatment caused a significant decrease in ultimate strength, in which ABX-TAC mice had the lowest ultimate strength among the four groups **(Figure 2C)**. Similar changes were observed in toughness and fracture strain, indicating that ABX treatment aggravated the loss of tensile strength and toughness, making the hearts more brittle and unable to absorb the energy without rupture **(Figure 2D** and **2E)**. Interestingly, we found a significant increase in compressive toughness in healthy ABX mice compared to healthy water controls **(Figure S3A** and** S3E)**. However, this effect was eliminated when subjected to TAC surgery, which decreased toughness, strength, and even stiffness under compression** (Figure S3B**-**S3E)**. This implies that the loss of tensile strength could be compensated by the gain in compressive strength under healthy control conditions but this was disabled under hypertrophic stress.

Since the mechanical strength of organs is mainly provided by the ECM, we sought to investigate further whether the loss of strength and elasticity was caused by modification in cardiac ECM. Collagen is the most abundant structural protein in cardiac ECM; therefore, we used picrosirius red staining to visualize the extent of collagen deposition. We found a significantly larger fibrotic area in ABX-TAC mice compared to Ctrl-TAC mice, while there was no difference between sham-operated groups **(Figure 2F**-**2H)**. Fibrotic area in TAC mice was positively correlated to cardiac catheter-derived diastolic function indicator- EDPVR slope, which was in turn positively correlated with tensile test-derived stiffness **(Figure 2I** and **2J)**. Collagen turnover during cardiac remodeling is chiefly regulated by myofibroblasts. Therefore, we examined myofibroblast abundance by quantifying vimentin and alpha-smooth muscle actin (α-SMA) double-positive cells. We found more myofibroblasts induced by TAC in the heart of ABX mice than in controls, while the increase in fibroblasts after TAC surgery was similar in these two groups **(Figure 2K**-**2M)**. This finding suggests that excessive fibrosis in ABX mice after TAC surgery could be attributed to hyperactivation of myofibroblasts. Apart from the abundance of collagen, the structure and composition of cardiac ECM are determinants of heart mechanics and organ strength as well. From the picrosirius red-stained sections, we noticed the presence of small collagen fiber fragments in the perivascular area **(Figure 2G)**. Therefore, we further looked into the ultrastructure of collagen fibers and found that collagen fibers were disarranged in ABX groups regardless of surgical type **(Figure 2N)**. Histogram of angle distribution shows that a bundle of collagen fibrils exhibits a predominant orientation with a wavy structure formed by fibrils which typically fall within a 60-degree angle of the dominant direction (assigned to be 0 degrees) in control mice, whereas collagen fibrils in the dominant direction have a lower frequency in both ABX groups **(Figure 2O)**. Using the orientation correlation function, we quantified the level of fiber alignment and found that both ABX groups had less aligned collagen fibrils compared to their water control counterparts **(Figure 2P)**. In addition, we noticed breakages and fragmentation of elastic fiber in the external elastic lamella in both ABX groups but not in control groups, suggesting that arterial elasticity might be altered in ABX mice **(Figure S4)**. Together, these data indicate that pre-depletion of gut microbiota with ABX treatment worsens LV stiffening and makes it more brittle after TAC surgery, which is associated with exacerbated collagen deposition and myofibroblast formation, accompanied by collagen fibrillar disarray.

### Normal gut microbiome benefits heart function by limiting cardiac ECM deposition and maintaining its structure

**Figure 3:**
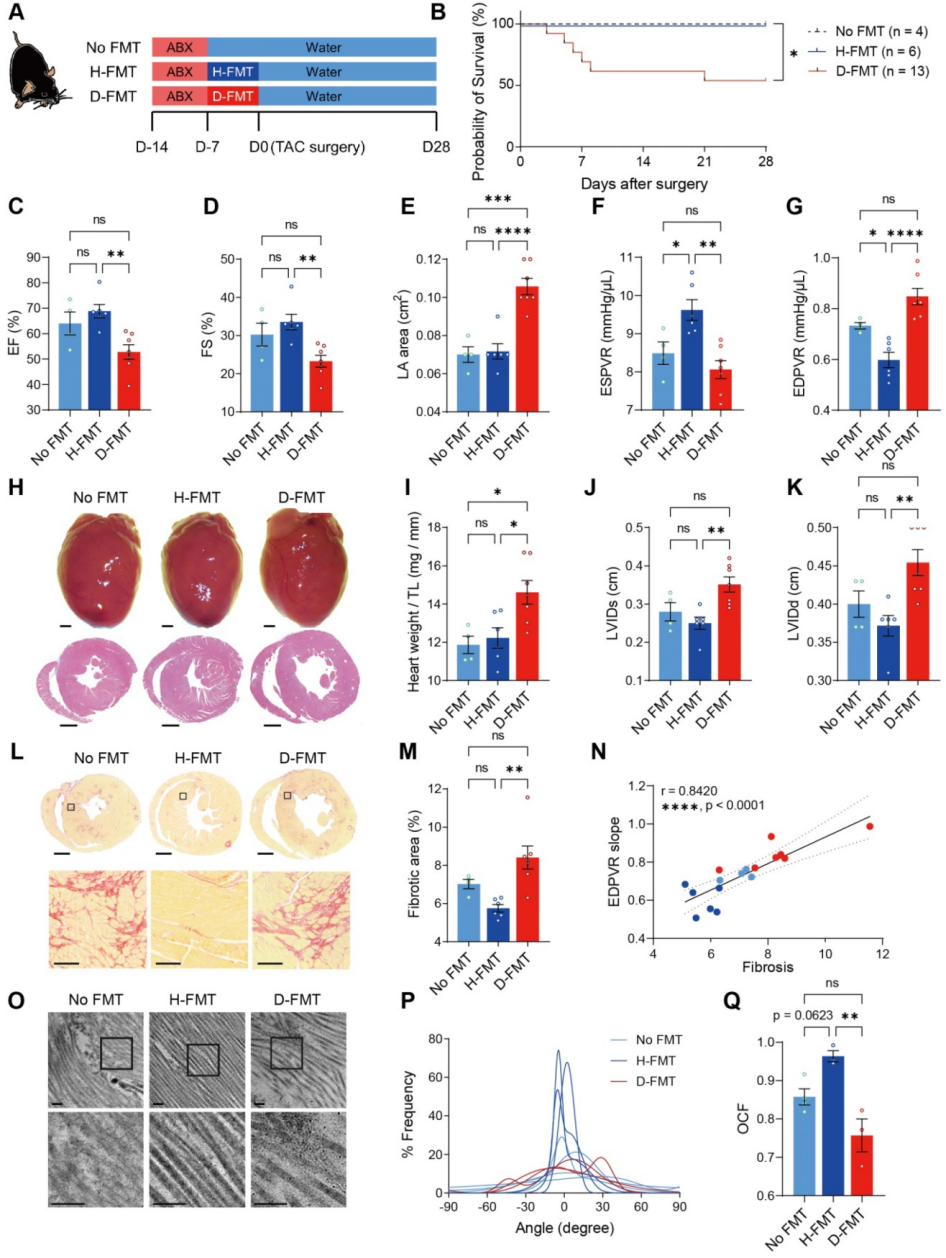
** Construction of healthy gut microbiome benefits heart function and ECM in SPF ABX mice after TAC surgery. A**, Experimental design for examining the effect of gut microbiota reconstruction on mice with dysbiosis after transverse aortic constriction (TAC) surgery. **B**, Probability of survival in mice receiving no fecal microbiota transplantation (FMT), healthy-FMT (H-FMT), and diseased-FMT (D-FMT). **C** through **E**, Heart systolic function was estimated by ejection fraction (EF) (**C**) and fractional shortening (FS) (**D**); diastolic function was estimated by left atrial (LA) area (**E**). **F** and **G**, Cardiac catheterization-derived pressure-volume relationship at end-systole (ESPVR) (**F**) and end-diastole (EDPVR) (**G**) measuring cardiac contractility and myocardial passive stiffness, respectively. **H**, Representative bright-field images of freshly isolated hearts and hematoxylin and eosin stained heart sections. Scale bar, 1 mm. **I**, Cardiac hypertrophy was assessed by heart weight normalized by tibial length (TL). **J** and **K**, Ventricular dilation was assessed by left ventricular internal dimensions at end-systole (LVIDs) (**J**) and end-diastole (LVIDd) (**K**). **L** and **M**, Representative images of picrosirius red-stained heart sections; upper scale bar, 1 mm; lower scale bar, 100 µm (**L**) and quantification of cardiac fibrotic area (**M**). **N**, Relation between EDPVR slope and fibrosis determined by simple linear regression. The solid line indicates the best-fit line, and the dashed lines above and below it indicate the upper and lower limit of the 95% confidence intervals of the linear regressions, respectively, and r is the correlation coefficient. **O**, Representative transmission electron microscope images showing collagen fibrils in mouse hearts. Scale bar, 0.2 µm. **P**, Collagen fibril angular distribution. **Q**, Orientation correlation function (OCF) for quantification of fiber alignment. The Kaplan-Meier method and the log-rank (Mantel-Cox) test were used to analyze the survival curves in **B**. Statistical significance was determined by ordinary one-way ANOVA with Tukey multiple comparisons test in **C** through **G**, **I**, **J**, **K**, **M**, and **Q**. ns, not significant; *, *P* < 0.05; **, *P* < 0.01; ***, *P* < 0.001; ****, *P* < 0.0001.

To verify that heart dysfunction and adverse ECM remodeling were induced by the absence of gut microbiota, we established a germ-free mouse model. To further test our hypothesis that commensal gut microbiota is required for heart remodeling under stress, we inoculated germ-free mice with cecal contents extracted from healthy donor mice (Ctrl-Sham mice); to examine the effects of heart disease-induced dysbiosis, we inoculated another group of germ-free mice with cecal contents obtained from donor mice subjected to TAC surgery for 28 days (Ctrl-TAC mice). After establishing gut flora, mice were subjected to surgery for induction of cardiac hypertrophy. However, because TAC surgery caused high mortality (two out of three mice in our preliminary test) in diseased FMT mice, we took an alternative approach to induce hypertrophic stress by using Angiotensin-II (Ang-II) infusion **(Figure S5A)**. Twenty-eight days of Ang-II infusion caused cardiac dysfunction in germ-free mice without FMT (no FMT), as evidenced by a decrease in cardiac output, increase in the left atrial area, and elevation in myocardial performance index **(Figure S5B-S5D)**. Transplantation of gut microbiota from healthy mice (H-FMT) ameliorates disease progression by preserving cardiac output, left atrial size as well as the myocardial performance index (**Figure S6**). However, transplantation of gut microbiota from diseased mice (D-FMT) showed no difference to no FMT mice, indicating that diseased gut microbiota had no beneficial effect on cardiac function. Moreover, the ESPVR slope was higher in healthy FMT mice compared to no FMT mice, indicating that a healthy gut microbiome restored heart contractility **(Figure S5E)**. In addition, the EDPVR slope tended to be lower in healthy FMT compared to no FMT mice, suggesting LV stiffening in hypertrophic hearts was attenuated by healthy FMT **(Figure S5F)**. There was no difference in either parameter between diseased FMT and no FMT mice, asserting that diseased gut microbiota was ineffective in improving heart function. To determine the level of cardiac hypertrophy, we weighed the hearts on day 28 and performed histological analysis. Surprisingly, we found that healthy FMT hearts were heavier than no FMT hearts **(Figure S5G** and **S5H)**. However, the decrease in LV internal diameters was minor in healthy FMT hearts **(Figure S5G**, **S5I** and** S5J)**. This suggests that healthy FMT hearts might undergo physiological hypertrophy with normal cardiac structure and preserved cardiac function. To further investigate potential differences in cardiac ECM and heart mechanics, we tested fibrosis and collagen ultrastructure and found that healthy FMT mice tended to have less fibrotic area compared to mice with no FMT, whereas diseased FMT mice had more severe fibrosis **(Figure S5K** and **S5L)**. Fibrosis was positively correlated to the catheter-derived index of LV stiffening **(Figure S5M)**. Furthermore, collagen fibrillar disarrangement was attenuated by healthy FMT but not diseased FMT **(Figure S5N-S5P)**. These data indicate that transplanting healthy gut microbiota into germ-free mice ameliorates adverse remodeling of cardiac ECM under hypertrophic stress, which leads to adaptive hypertrophy with preserved heart function and collagen structure and amount. We then used specific pathogen-free (SPF) mice to confirm the effect of healthy gut microbiota in the TAC model. Mice were deprived of their intrinsic gut microbiota using ABX followed by gut microbiota transplantation to establish the desired gut microbiome before surgery **(Figure 3A)**. The reconstitution of dominant gut microbes in healthy and diseased donors, respectively, in the recipients were confirmed to have comparable total abundance and similar trends of difference between the two groups (**Figure S7A**-**S7L**). Notably, Bacteroides thetaiotaomicron became the dominant species replacing Muribaculum intestinale in both groups of recipients, with the two bacterial species having positive correlations in both healthy and diseased donors (**Figure S7M** and **S7N**). Linear discriminant effect size (LEfSe) analysis shows that species enriched in healthy donors were Roseburia intestinalis, Acutalibacter muris, Paludicola psychrotolerans, and Anaerotaenia torta; whereas Eubacterium coprostanoligenes, Parabacteroides goldsteinii, Bacteroides thetaiotaomicron, Akkermansia muciniphila, and Prasutterella excrementihominis were more abundant in diseased donors (**Figure S7O**). Among the 11 core gut microbes identified from the 16S sequencing result of healthy and diseased donors, Pseudoflavonifractor capillosus, Lachnoclostridium pacaense, Roseburia intestinalis, and Anaerocolumna cellulosilytica were found enriched in H-FMT recipients; none of the core species were significantly more abundant in D-FMT mice (**Figure S7P**). Similar to the germ-free mouse model, we found a high mortality in diseased FMT mice **(Figure 3B)**. Compared to diseased FMT mice, healthy FMT mice had attenuated heart dysfunction after TAC surgery, as shown by higher EF and FS with a smaller left atrial area **(Figure 3C-3E)**. In addition, heart contractility and elasticity were higher in healthy FMT mice compared to diseased FMT mice **(Figure 3F** and **3G)**. Cardiac hypertrophy and LV dilation were attenuated in healthy FMT compared to diseased FMT mice, with no FMT having a milder effect **(Figure 3H-3K)**. However, ECM amount and structure could only be rescued by healthy FMT but not diseased FMT or residual microbiota recovered from ABX treatment **(Figure 3L-3Q)**. Taken together, these data confirmed that the cause of worsened heart dysfunction and ECM adverse remodeling under hypertrophic stress was the lack of gut microbiota instead of ABX cardiotoxicity, which could be reversed by the construction of gut flora obtained from healthy donors but not diseased donors.

### Acetate and propionate are enriched products in a healthy microbiota

**Figure 4:**
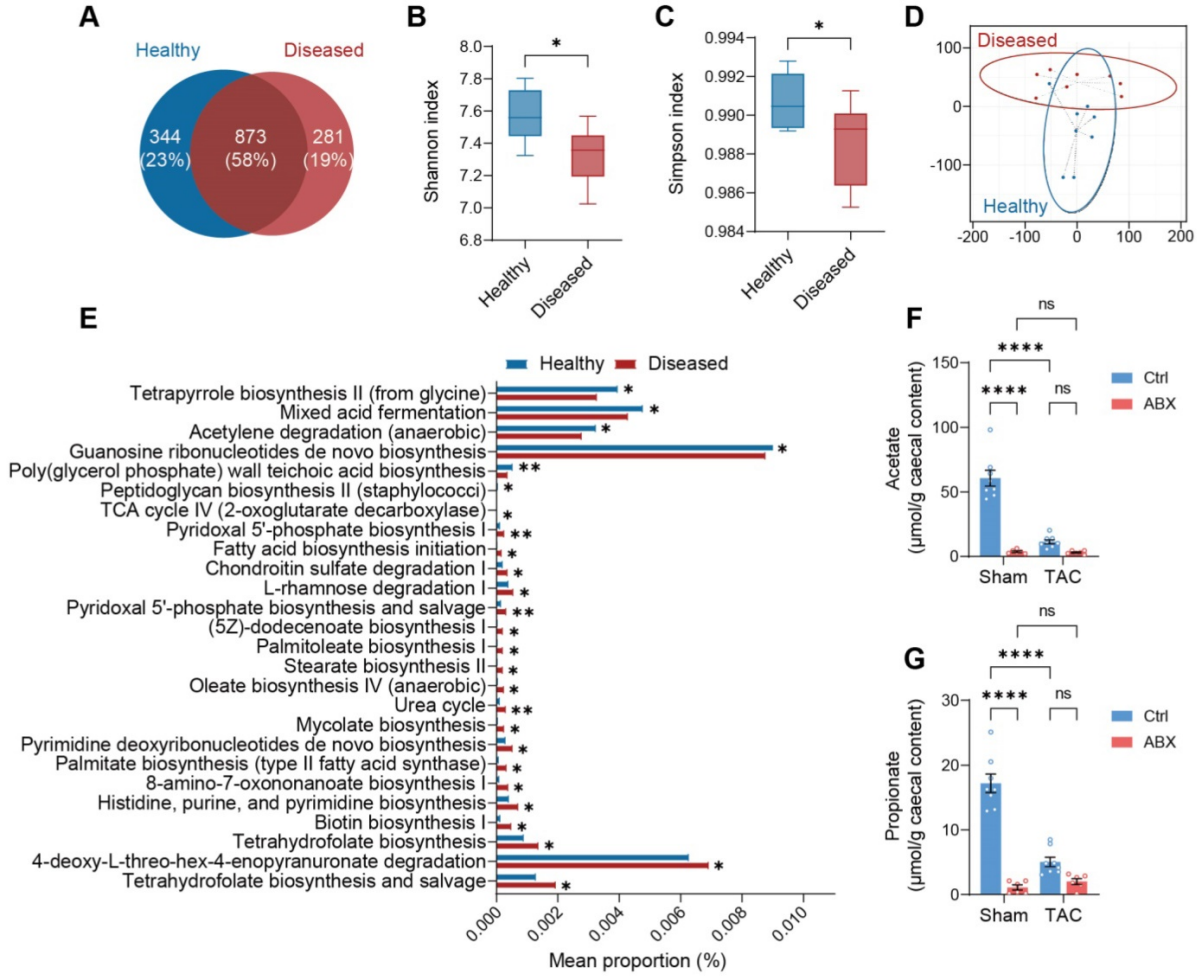
** Healthy gut microbiota is distinguished from diseased gut microbiota by greater production of acetate and propionate. A**, Venn diagram of gut microbial species in the cecal contents extracted from healthy and diseased donors. **B** and **C**, Alpha diversity analysis showing richness and evenness of the gut microbiota was estimated by Shannon index (**B**) and Simpson index (**C**). Statistical significance was determined by an unpaired two-tailed t-test, *, *P* < 0.05. **D**, Beta diversity shown by weighted UniFrac t-distributed stochastic neighbor embedding analysis. **E**, Phylogenetic Investigation of Communities by Reconstruction of Unobserved States analysis showing pathways differentially enriched in the healthy and diseased donors. **F** and **G**, Quantification of acetate (**F**) and propionate (**G**) in the cecal contents of donors for microbiota transplantation experiments and antibiotics-treated mice. Statistical significance was determined by two-way ANOVA with Tukey multiple comparisons test. ns, not significant; ****, *P* < 0.0001.

Next, we sought to investigate the difference between the healthy microbiota (extracted from cecal contents of Ctrl-Sham mice) with beneficial effects and the ineffective diseased microbiota (extracted from cecal contents of Ctrl-TAC mice). We sequenced the microbiota extracted from the cecal contents of both groups of donors and found that nearly 25% of the healthy bacteria were replaced by other species in the diseased state **(Figure 4A)**. From graphical phylogenetic analysis, we found that healthy and diseased mice shared 16 gut microbes out of the top 18 microbiota that cover over 44% of all species. The shared microbes were Muribaculum intestinale, Anaerocolumna cellulosilytica, Eisenbergiella massiliensis, Eisenbergiella tayi, Faecalicatena contorta, Kineothrix alysoides, Lachnoclostridium pacaense, Lacrimispora saccharolytica, Roseburia faecis, Oscillibacter valericigenes, Acutalibacter muris, Anaerotruncus rubiinfants, Flavonifractor plautii, Phocea massiliensis, Pseudoflavonifractor capillosus, and Desulfovibrio desulfuricans. The dominant gut microbiota unique to healthy mice were Acetatifactor muris and Lacrimispora aerotolerans; whereas those unique to diseased mice were Bacteroides thetatiotaomicron and Enterocloster bolteae (**Figure S8**). Alpha-diversity, indicating richness and evenness, was higher in healthy donors **(Figure 4B** and **4C)**. On the other hand, beta-diversity, measured by weighted UniFrac t-distributed stochastic neighbor embedding (t-SNE), showed that the two communities were divergent **(Figure 4D)**. Phylogenetic Investigation of Communities by Reconstruction of Unobserved States (PICRUSt) analysis revealed that the healthy community enriched mixed acid fermentation and acetylene degradation which produce acetate, succinate, and lactate, with the latter two being the precursors of propionate 17, 18 **(Figure 4E** and **Table S1)**. To confirm that the healthy donors favor acetate and propionate production, we tested the abundance of these metabolites in the cecal contents of Ctrl-Sham (i.e., the healthy microbiota donors) and Ctrl-TAC (i.e., the diseased microbiota donors) as well as ABX-Sham and ABX-TAC mice. We found that the concentration of both acetate and propionate was significantly lower in diseased donors compared to healthy donors **(Figure 4F** and **4G)**. Unsurprisingly, both ABX groups showed low acetate and propionate levels, which could be attributed to the depletion of gut microbiota. We also confirmed the circulating acetate and propionate levels in the mice and found the same trend as in cecal contents (**Figure S9**). Furthermore, metabolites involved in the mixed acid fermentation and acetylene degradation were measured and a trend of decrease was seen in all metabolites in diseased donors compared to healthy donors (**Figure S10**). Interestingly, in germ-free mice transplanted with healthy gut microbiota, Ang-II tended to lower fecal acetate and propionate levels, which is similar to the effect of TAC on cecal acetate and propionate in SPF mice (**Figure S11**).

### Acetate and propionate rescue heart function and ECM disarray under hypertrophic stress

**Figure 5:**
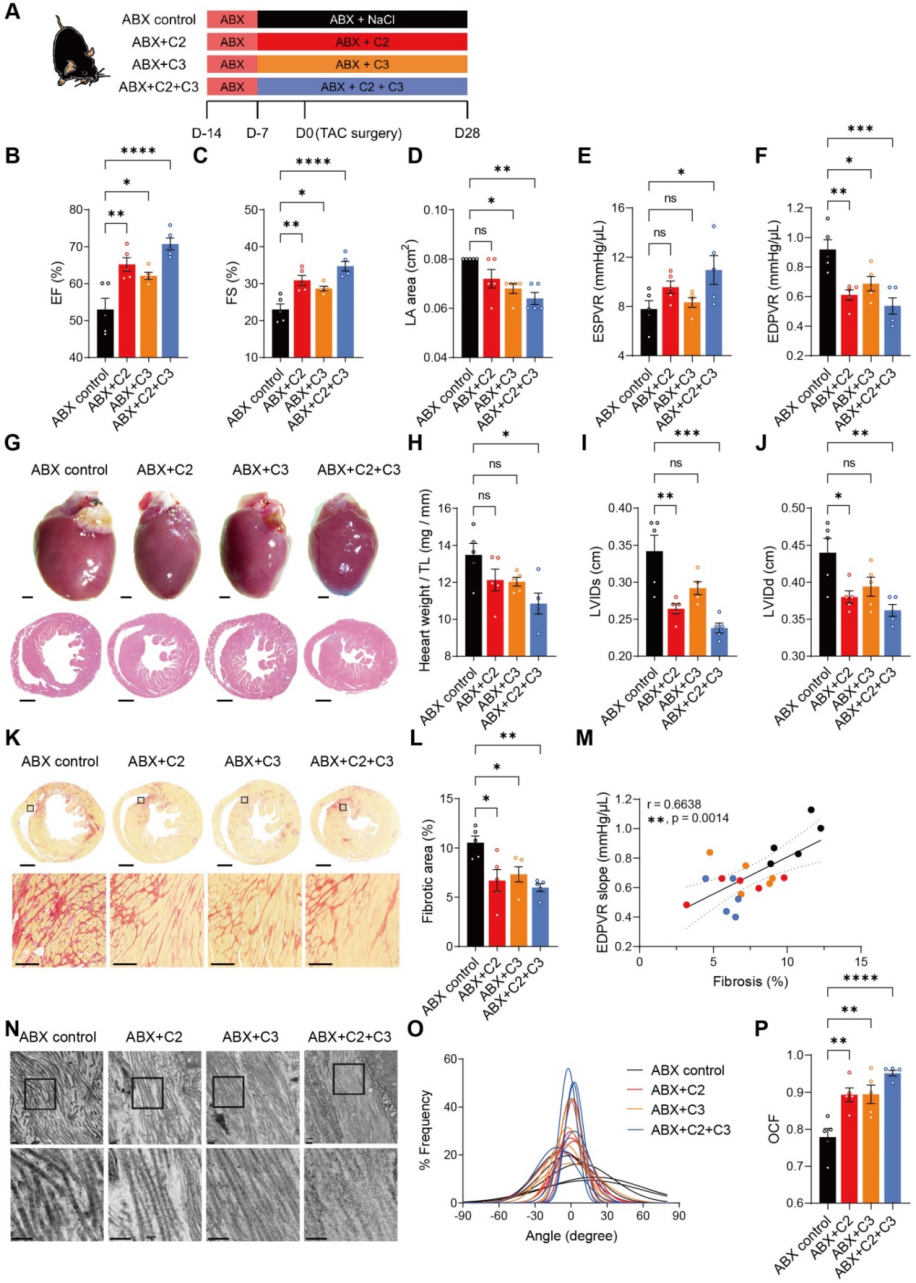
** Supplementation of acetate and propionate rescues cardiac outcomes and collagen disarray after transverse aortic constriction. A**, Experimental design for examining the effect of acetate (C2) and propionate (C3) on mice with dysbiosis after transverse aortic constriction (TAC) surgery. **B** through **D**, Heart systolic function was estimated by ejection fraction (EF) (**B**) and fractional shortening (FS) (**C**); diastolic function was estimated by left atrial (LA) area (**D**). **E** and **F**, Cardiac catheterization-derived pressure-volume relationship at end-systole (ESPVR) (**E**) and end-diastole (EDPVR) (**F**) measuring cardiac contractility and myocardial passive stiffness, respectively. **G**, Representative bright-field images of whole hearts and hematoxylin and eosin stained heart sections. Scale bar, 1 mm. **H**, Cardiac hypertrophy was assessed by heart weight normalized by tibial length (TL). **I** and **J**, Ventricular dilation was assessed by left ventricular internal dimensions at end-systole (LVIDs) (**I**) and end-diastole (LVIDd) (**J**). Representative images of picrosirius red-stained heart sections; upper scale bar, 1 mm; lower scale bar, 100 µm (**K**) and quantification of cardiac fibrotic area (**L**). **M**, Relation between EDPVR slope and fibrosis determined by simple linear regression. The solid line indicates the best-fit line, the dashed lines above and below it indicates the upper and lower limit of the 95% confidence intervals of the linear regressions, respectively, and r is the correlation coefficient. **N**, Representative transmission electron microscope images showing collagen fibrils in mouse hearts. Scale bar, 0.2 µm. **O**, Collagen fibril angular distribution. **P**, Orientation correlation function (OCF) for quantification of fiber alignment. Statistical significance was determined by ordinary one-way ANOVA with Tukey multiple comparisons test in **B** through **F**, **H**, **I**, **J**, **L**, and **P**. ns, not significant; *, *P* < 0.05; **, *P* < 0.01; ***, *P* < 0.001; ****, *P* < 0.0001.

To test whether acetate (C2) and propionate (C3) exert beneficial effects despite depletion of gut microbiota on the recovery of heart function and ECM, we supplemented ABX mice with these metabolites **(Figure 5A)**. At termination, the success of supplementation was confirmed by higher acetate levels in cecal contents and circulation in all C2-receiving mice (ABX+C2 and ABX+C2+C3) compared to ABX control mice (**Figure S12A** and **S12C**); and higher cecal and circulating propionate levels in C3-receiving groups (ABX+C3 and ABX+C2+C3) compared to the control (**Figure S12B** and **S12D**). Four weeks after TAC surgery, C2 and C3 supplemented mice had significantly enhanced cardiac function, with combined treatment giving rise to even better outcomes **(Figure 5B**-**5D)**. In addition, improvement in ventricular contractility and elasticity was achieved by C2 and C3 supplementation **(Figure 5E** and **5F)**. Cardiac hypertrophy estimated by histology and heart weight were significantly attenuated by C2 and C3 combined treatment **(Figure 5G** and **5H)**. Similarly, LV dilation was less severe in C2 and C3 treated mice** (Figure 5I** and** 5J)**. Adding C2 and C3 effectively ameliorated the processes of cardiac ECM remodeling disturbed by the absence of gut microbiota, with combined treatment significantly reducing fibrotic area and preventing collagen fibrillar disarray **(Figure 5K-5P)**. Together, these results demonstrate that the efficacy of healthy gut microbiota in benefiting heart function and mechanics was mainly achieved by producing acetate and propionate, which produce protective effects comparable to normal gut flora.

### Acetate and propionate prevent SMAD2 activation under fibrogenic stress through GPR41 and GPR43 in cardiac fibroblasts

**Figure 6:**
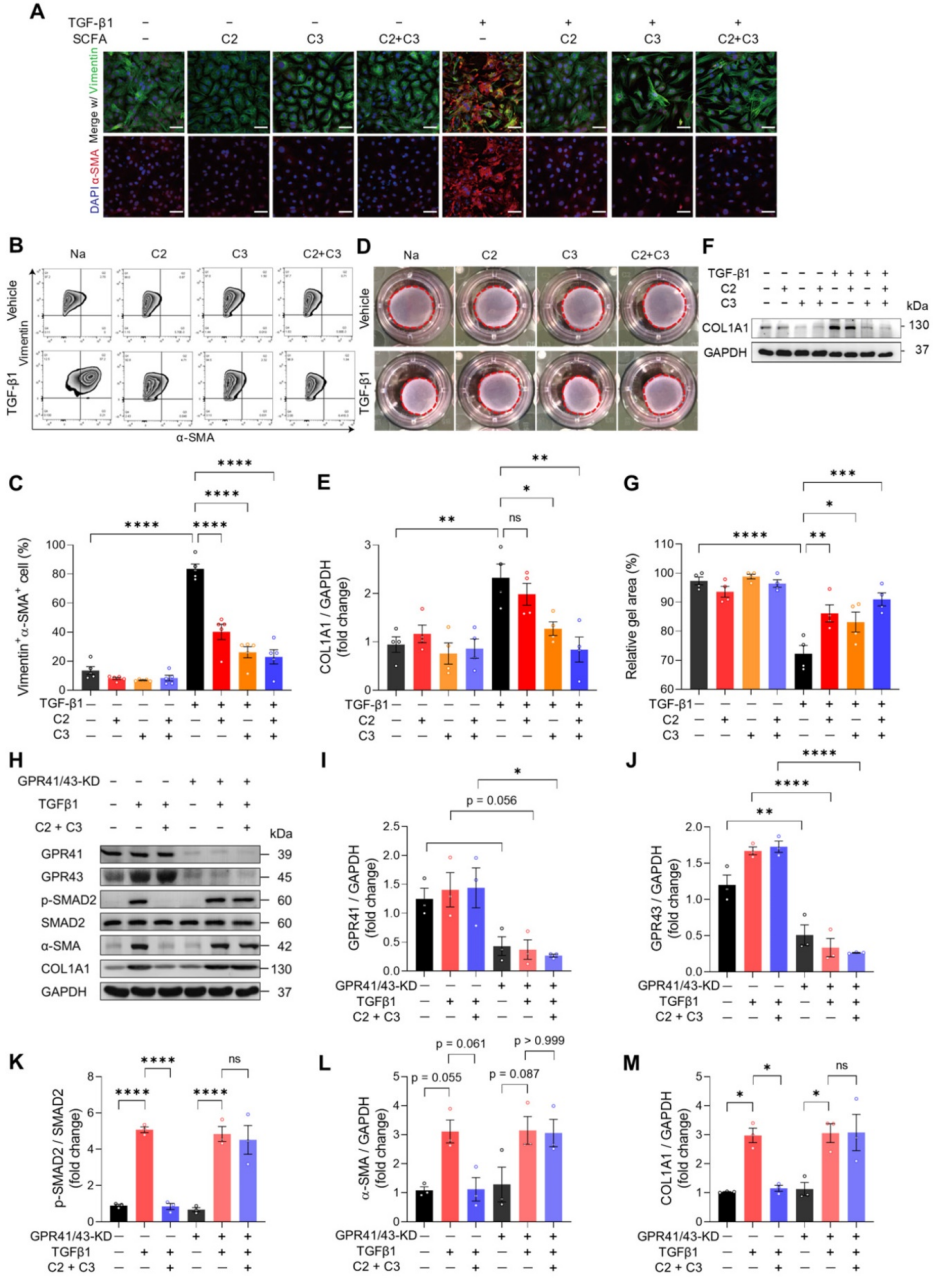
** Acetate and propionate prevent myofibroblast transition from cardiac fibroblasts through G-protein coupled receptor 41 and 43. A**, Immunofluorescent staining of vimentin and alpha-smooth muscle actin (α-SMA) expression in mouse primary cardiac fibroblasts treated with acetate (C2) and propionate (C3) alone or in combination (C2+C3) to identify myofibroblast formation induced by transforming growth factor beta 1 (TGF-β1). Scale bar, 50 µm. **B** and **C**, Representative flow cytometry results of cardiac fibroblasts expressing vimentin and α-SMA (**B**) and quantification of vimentin and α-SMA double-positive cells (**C**). **D** and **E**, Representative images of collagen gels for assessing cardiac fibroblast contractility (**D**) and quantification of gel area (**E**). **F** and **G**, Representative images of Western blot performed to identify collagen formation with TGF-β1 fibrogenic stimulus in cardiac fibroblasts treated with C2 and C3 (**F**) and quantification of collagen expression (**G**). **H** through **M**, Western blotting of proteins involved in the TGF-β1-induced fibrogenesis pathway in GPR41 and GPR43 double knockdown cardiac fibroblasts (**H**) with quantification of GAPR41 (**I**), GPR43 (**J**), p-SMAD2 (**K**), α-SMA (**L**), and COL1A1 (**M**) protein expression. Statistical significance was determined by ordinary one-way ANOVA with Tukey multiple comparisons test in **C**, **E**, **G**, **I**, **J**, **K**, **L**, and **M**. ns, not significant; *, *P* < 0.05; **, *P* < 0.01; ***, *P* < 0.001; ****, *P* < 0.0001.

To investigate how acetate and propionate participate in cardiac ECM remodeling which ultimately benefits heart functioning, we isolated mouse cardiac fibroblasts and treated them with C2, C3, or combined. TGF-β1 was used to induce fibrogenic stress. The mRNA expression of fibrogenic genes was increased by TGF-β1 and this was reversed by C2 and C3 alone and combined treatment **(Figure S13A-S13C)**. We then stained cardiac fibroblasts with vimentin and α-SMA to visualize activated fibroblasts and found that TGFβ1-induced myofibroblast transition was reversed by C2, C3, and combined treatment **(Figure 6A)**. The finding was further confirmed by quantifying vimentin and α-SMA double-positive cells using flow cytometry **(Figure 6B** and **6C)**. Moreover, the increase in cardiac fibroblast contractility, measured by collagen contraction assay, induced by TGF-β1 was attenuated by C2, C3, and combined treatment **(Figure 6D** and **6E)**. This suggests that tissue stiffening, which is positively correlated to high fibroblast contractility, could be reversed by C2 and C3. To test if the reduction of activated cardiac fibroblasts in C2 and C3 treated groups inhibit collagen deposition, we examined the protein expression of COL1A1, the major component of cardiac ECM, and found that TGF-β1-induced collagen deposition was prevented by C3 and more significantly by C2 and C3 combination **(Figure 6F** and **6G)**. To elucidate the molecular mechanism underlying the anti-fibrogenic effect of acetate and propionate, we knocked down their receptors, GPR41 and GPR43 on mouse primary cardiac fibroblasts to test if they function through these receptors 19. The results showed that C2 and C3 inhibit SMAD2 activation, which suppresses the pro-fibrogenic effect of TGFβ1. This phenomenon was blunted in GPR41/GPR43-KD cardiac fibroblasts, in which C2 and C3 treatment have no effect on suppressing SMAD2 phosphorylation. To confirm that C2- and C3-induced anti-fibrogenesis in GPR41/GPR43-KD fibroblasts was impeded, we assayed α-SMA and COL1A1 expression and found no suppression of these proteins by C2 and C3 in GPR41/GPR43-KD fibroblasts **(Figure 6H**-**6M)**. Taken together, these data identified acetate and propionate as suppressing fibrogenesis under stress via inhibiting SMAD2 activation through GPR41 and GPR43 receptors on cardiac fibroblasts.

## Discussion

The gut microbiota has been implicated in an increasing variety of cardiovascular diseases including cardiac rupture after myocardial infarction 4. However, the cause of the structural modification has not been previously explored. Here, we showed that the gut microbiota, through the production of acetate and propionate, prevents cardiac fibroblast activation and thus averts excessive fibrosis. In addition, cardiac ECM structure was maintained by normal gut microbiota, which might be attributed to higher ventricular elasticity, tensile strength, and toughness to promote heart structural integrity and protect it from rupture.

We found that ABX treatment, which abolishes the vast majority of gut microbiota and the derivative acetate and propionate, exacerbates cardiac function after TAC-induced cardiac hypertrophy. This was further validated by germ-free mice with Ang-II-induced hypertrophy. Using two types of animal models (germ-free and SPF), and two cardiac stress models (TAC and Ang-II), with two different readouts (EF or myocardial performance index and hemodynamic parameters), we concluded that the absence or imbalance of gut microbiota is detrimental to heart mechanics. Of note, in SPF mice with dysbiosis, the no FMT group served as the negative control of microbiota transplantation, but it was dissimilar to germ-free mice with no FMT, which were completely free from microbiota. The residual gut microbiota in the SPF no FMT model might be replenished during the five weeks after cessation of ABX treatment before sacrifice, which may account for the milder cardiac dysfunction seen in the no FMT SPF mice compared to diseased-FMT SPF mice.

Regarding the SPF ABX mouse model, it has been shown that ABX treatment can benefit cardiac outcomes after TAC surgery by hampering T cell activation 20, which might indicate that a high dosage of ABX (four-fold concentration in the current study) could exert T-cell inhibition that outweighs the effect of ECM adverse remodeling caused by dysbiosis due to low dosage of ABX treatment. In addition, the beneficial effect of short-chain fatty acids in attenuating hypertrophy has been reported previously. For example, it has been shown that supplementation of propionate attenuated cardiac hypertrophy and fibrosis in Ang-II-infused wild-type and ApoE knockout mice by reducing splenic T cells 21. Displaying the consistent results that propionate ameliorates adverse remodeling, the previous study focused on the anti-inflammatory role of propionate, while our current study displayed its benefit from a new point of view by exploring the biomechanics of hearts and unveiling the direct interaction with cardiac fibroblasts.

Using both cardiac catheterization and tissue-level mechanical tests, we found that both TAC and ABX treatment cause heart stiffening and loss of tensile strength, which leads to exacerbated mechanical modifications in ABX-TAC mice. It has been reported that heart failure, induced by TAC or Ang-II, changes heart mechanics by myocardial stiffening 22, 23, which is consistent with our findings that ABX mice have more severe cardiac hypertrophy and dysfunction with more intensive myocardial stiffening.

In both normal and diseased myocardium, fibrillar collagens are the major contributors responsible for the maintenance of tissue structure 24. In our animal models of dysbiosis, we observed excessive collagen synthesis and accumulation in a disorganized manner. These modifications in collagen were shown to be correlated to impaired heart mechanical properties and heart failure in various animal models and clinical studies 25, 26. Apart from the increase in amount, the orientation or alignment of collagen in determining cardiac function has garnered attention recently. For example, researchers found that deficiency in collagen V causes fibrillar collagen disarray and ultimately leads to worsened cardiac outcome 27.

To test the difference between the healthy and diseased gut microbiome that leads to divergent disease outcomes, we analyzed the components in each bacterial community. As the healthy and diseased donors shared 16 out of the 18 top gut microbes, most of which produce acetate and/or propionate, the association between acetate/propionate production and dominant gut microbial species was unclear. For example, Pseudoflavonifractor capillosus produces acetate and succinate 28, Lacrimispora saccharolytica and Anaerocolumna cellulosilytica produce acetate 29, 30, and the major fermentation products of Flavonifractor plautii are propionate and butyrate 31. However, as the function of gut microbiota is altered by culture conditions, the production of these short-chain fatty acids may vary in different gut environments. Of the gut microbes dominant in healthy donors, Acetatifactor muris produces acetate and propionate 32, and Lacrimispora aerotolerans also produces acetate 33. Similarly, gut microbes dominant in diseased donors, Bacteroides thetatiotaomicron and Enterocloster bolteae, also produce acetate and propionate 34, 35. However, the gut microbes dominant in the healthy group were also found in the diseased group, and vice versa. Of other gut microbes that were more abundant in healthy donors, Lacrimispora algidixylanolytica produces acetate and lactate 36, and Roseburia intestinalis produces lactate 37. Therefore, we tried to explore the different effects of the gut microbiota as a whole instead of according to individual species. Interestingly, Roseburia intestinalis, an acetate-consumer, was only found in healthy donors, i.e., none of the diseased donors possess this gut microbe. This indicates that the diseased gut environment, having significantly lowered acetate, was unsuitable for the growth of Roseburia intestinalis. To understand the colonization of healthy and diseased gut microbiota, further investigations of the microbial composition in FMT recipients would be important for subsequent studies. We also observed less richness of diseased gut microbiota, which was correlated to higher cardiovascular disease risk in a US cohort 38. Interestingly, low bacterial richness has also been linked to obesity and inflammatory phenotypes 39, which are risk factors for cardiovascular diseases. Moreover, low bacterial richness could be corrected by dietary intervention that shifts metabolic homeostasis towards better status with less circulating cholesterol, inflammation, and adiposity 40. Our current results showing low bacterial richness in diseased microbiota donors, characterized by low acetate and propionate levels, are in line with these reports.

Lastly, the *in vitro* experiment using mouse primary cardiac fibroblasts treated with acetate and propionate demonstrated the direct anti-fibrogenic efficacy of acetate and propionate in cardiac fibroblasts. Indeed, the effect of short-chain fatty acids (mostly butyrate) in inhibiting fibroblast activation has been reported in human fetal lung fibroblasts 41. However, there is no study focusing on the direct effect specifically for primary cardiac fibroblasts. In light of the finding that acetate and propionate inhibit TGF-β1 induced fibrogenesis, we further explored the canonical downstream SMAD2 phosphorylation which has been reported to upregulate α-SMA and COL1A1 expression in myofibroblasts and other cell types 42-44. We demonstrated that the downstream SMAD2 signaling pathway was obstructed. In addition, knockdown of GPR41 and GPR43 in cardiac fibroblasts disabled acetate and propionate from inhibiting SMAD2 activation and therefore invalidated the function of the metabolites on the GPR41/GPR43-SMAD2 pathway. The association between acetate and TGFβ/SMAD2 pathway has been reported through AMPK-c-Jun pathway in hepatic stellate cells 45. To further investigate the underlying mechanism, we tested if c-Jun and AMPK phosphorylation was affected by acetate and propionate treatment. Unfortunately, the expression of c-Jun and AMPK were unaffected by acetate and propionate, regardless of the presence of GPR41 and GPR43 receptors (**Figure S14**). It would be important for subsequent studies to explore other signaling pathways by which acetate and propionate suppress SMAD2 phosphorylation and fibrotic protein expression.

Through this work, we identified a previously undescribed role of gut microbiota-derived acetate and propionate in inhibiting TGF-β1-SMAD2 signaling pathways in cardiac fibroblasts via GPR41 and GPR43 receptors. We showed that the normal gut flora, through producing acetate and propionate, controls myofibroblast transition from cardiac fibroblasts that protect hearts from excessive fibrosis under stress and endows hearts with normal ECM fibrous geometry and mechanical strength. In contrast, depletion of gut microbiota or reconstruction of the diseased gut microbiome accelerates disease progression by excessive fibrogenesis, ventricular stiffening, and ECM fibrillar disarray. These findings highlight the newly-identified role of gut microbiota-derived metabolites in maintaining cardiac ECM homeostasis and heart mechanics through cardiac fibroblasts.

## Supplementary Material

Supplementary methods, figures and table.
