# Supplementary material for: Commensal gut microbiota-derived acetate and propionate enhance heart adaptation in response to cardiac pressure overload in mice
Source: Theranostics. 2022 Oct 17;12(17):7319–34. doi: 10.7150/thno.76002 (PMC9691357; doi:10.7150/thno.76002)
Supplement: Supplementary file 1 — Supplementary methods, figures and table. [file thnov12p7319s1.pdf]

Commensal gut microbiota-derived acetate and propionate enhance heart adaptation in response to cardiac pressure overload in mice

Chen-Ju Lin[1], Yu-Che Cheng[1], Hung-Chih Chen[1], Yu-Kai Chao[1], Martin W. Nicholson[1], Eric C.L. Yen[1,2], Timothy J. Kamp[3], Patrick C. H. Hsieh[1,3,4]

[1]Institute of Biomedical Sciences, Academia Sinica, Taipei 115, Taiwan
[2]Department of Nutritional Science, University of Wisconsin-Madison, Madison, WI 53705, USA
[3]Department of Medicine and Stem Cell and Regenerative Medicine Center, School of Medicine and Public Health, University of Wisconsin-Madison, Madison, WI 53705, USA
[4]Institute of Medical Genomics and Proteomics and Institute of Clinical Medicine, National Taiwan University, Taipei 106, Taiwan

Supplementary: Supplemental methods, 14 figures, 1 table

Correspondence:
Patrick C.H. Hsieh, MD, PhD
Institute of Biomedical Sciences, Academia Sinica
128 Academia Road, Section 2, Nankang District, Taipei 115, Taiwan
Phone: 886-2-27899074
Email: phsieh@ibms.sinica.edu.tw

Expanded methods

Quantitative PCR for ABX efficiency in depleting the gut microbiota

ABX efficiency in depleting gut microbiota was determined by quantifying 16S rRNA gene copy number by quantitative PCR using bacterial genomic DNA extracted from mouse cecal contents using innuSPEED stool DNA kit (Qiagen) with primers (forward: 5'-TCCTACGGGAGGCAGCAGT -3' and reverse: 5'- GGACTACCAGGGTATCTAATCCTGTT -3'). Each reaction for qPCR was prepared by mixing 4 µL 5X OmicsGreen qPCR MasterMix (Omics Bio), 1 µL 10 µM forward primer, 1 µL 10 µM reverse primer, 100 ng DNA template (concentration was determined by NanoDrop Spectrophotometer, ND-1000) with nuclease-free water to make a total volume of 20 µL. Reactions were performed using a real-time PCR system (ABI 7500, Applied Biosystems) with a thermal profile of 2 min 50 °C – 2 min 95 °C – 40 cycles of 15 s 95 °C and 1 min 50 °C, followed by a melt curve analysis performed with 60-95 °C.

Tensile and compressive tests for heart mechanical property measurement

Fresh heart samples were collected for testing right after sacrifice. Left ventricular tissues were dissected and trimmed to be dumbbell-shaped and the minimum cross-sectional area was estimated by averaging three measurements of the diameter. Samples were then applied to material testing machine in uniaxial tension at room temperature (RT). The test was started with the software program. The sample was loaded under tension to 30 N at 1 mm/s; a rate and load that do not cause failure of the tissue. Elastic property was derived from the force and length data acquired from the software. For the compressive test, freshly isolated heart samples were collected with their apex and atria removed to make two parallel surfaces. The samples were then placed onto the testing machine and subjected to 30 N load at 1 mm/s. Elastic property under compression was then determined by force and length data acquired from the software.

Transmission electron microscopy for cardiac collagen ultrastructure analysis

The apex of heart tissues was dissected into pieces of around 1 mm^3 for formalin fixation in addition with 2.5% v/v glutaraldehyde in 100 mM cacodylate buffer (pH 7.2) (wash buffer) for 2.5 h at RT. Samples were then washed for three changes for 5 min each at RT. Samples were incubated in 2% v/v osmium tetroxide with 1.5% w/v potassium ferrocyanide in wash buffer for 1 h at RT, followed by five washes with ddH_2O for 3 min each at RT. Samples were then incubated in 1% w/v tannic acid in wash buffer for 2 h at 4°C, followed by five washes with ddH_2O for 3 min each at RT. Samples were subjected to the second osmium staining for 40 min before uranyl acetate (1% w/v) staining overnight. After three washes for 5 min each, samples were dehydrated with increasing ethanol concentrations (30, 50, 70, 90, and 100% v/v) in ddH_2O using four washes of 10 min each at RT. Samples were transferred to transition solvent, propylene oxide, for 10 min at RT before being infiltrated in a graded series of resin

(Agar 100, Hard, Elektron Technology) (30% for 4 h, 50% overnight, and 70, 90, 100, 100, 100% for 1 h) in transition solvent at RT, and finally embedded in flat molds with fresh 100% resin for 72 h at 60°C. Embedded samples were then cooled and removed from the molds. The samples were sectioned to be 70-90-nm-thick using an ultramicrotome (EM UC7, Leica Microsystems), placed on copper grids, and stained with 1% w/v toluidine blue followed by rinsing in ddH_2O and drying. A transmission electron microscope (Tecnai G2 F20 S-TWIN, FEI) was used to image cardiac collagen fibers. Sections were observed at 80 kV accelerating voltage. Images were captured with Kodak 4489 negatives. At least three images were captured and analyzed for each animal. Analysis and quantification were performed by a blinded investigator different from the personnel capturing the images.

Histological analysis

The hearts harvested from both ABX and control mice with sham or TAC surgery were fixed in 4% paraformaldehyde (Sigma), paraffin-embedded and cut longitudinally into 5-μm sections. Deparaffinized serial heart sections were subjected to hematoxylin and eosin stain, picrosirius red stain, or Verhoeff-van Gieson stain following the manufacturer's instructions to visualize tissue morphology, collagen deposition, and elastic fiber morphology, respectively. The samples were examined using a Pannoramic 250 FLASH II slide scanner (3DHISTECH) or an Axio Imager.A1 microscope (Zeiss) and analyzed using the CaseViewer software v.2.2 (3DHISTECH). The fibrotic area was quantified using the ImageJ software v.1.53f51 (Rasband et al., NIH, USA). At least three images from the basal, middle, and apical sections, respectively, from each mouse, were captured and analyzed. Analysis and quantification were performed by a blinded investigator different from the person capturing the images.

Immunofluorescence staining for quantification of myofibroblast

The heart sections prepared as described in the "Histological Analysis" section were deparaffinized and cooked in 10 mM citric acid (pH 6.0) for 20 min by a pressure cooker. The heart sections were then blocked with 10% normal goat serum in PBST for 1 h at RT, incubated with mouse monoclonal anti-Vimentin (V2258, Sigma, 1:200) and rabbit polyclonal anti-α-SMA (ab5694, Abcam, 1:200) overnight at 4°C, washed and incubated with Alexa-Fluor-conjugated secondary antibodies against mouse IgM (A-10680, Invitrogen, 1:200) and rabbit IgG (A-11011, Invitrogen, 1:200) for 1 h at RT, and incubated with DAPI (D1306, Invitrogen, 1:2,500) for 10 min at RT. The samples were examined using a LSM 700 stage confocal microscope (Zeiss) and analyzed using the ZEN software (Zeiss). At least three images from the basal, middle, and apical sections, respectively, from each mouse, were captured and analyzed. Analysis and quantification were performed by a blinded investigator different from the personnel capturing the images.

Liquid chromatography–tandem mass spectrometry measurement of circulating acetate and propionate

Plasma samples were collected from the heart after mice were subjected to cardiac catheterization. Samples were stored at -80°C until thawing for liquid chromatography-tandem mass spectrometry analysis. 20 µL samples were mixed with 140 µL methanol using Geno/Grinder (Spex SamplePrep) and centrifuged at 18,000 × g for 10 min at 4°C. The supernatant was used for 3-NPH derivation and added with internal standards. The samples were then analyzed using Agilent 1290 II UHPLC coupled with an Agilent 6495c triple quadrupole mass spectrometer (Agilent Technologies) and a C18 column (2.1 x 100 mm, 1.8 µm) (ZORBAX Eclipse Plus, Agilent Technologies). 0.1% formic acid in water and 0.1% formic acid in IPA/ACN (3 to 1) were used as the mobile phase.

High-performance liquid chromatography measurement of acetate and propionate

Cecal contents and feces were collected, snap-frozen in liquid nitrogen immediately, and stored at -80°C, until thawing for high-performance liquid chromatography analysis. Before the experiment, cecal contents were thawed and homogenized in 1 mL of HPLC grade water per 50 mg sample and centrifuged at 17,000 × g. The supernatant was then passed through a 0.22-µm syringe filter to remove bacterial cells and debris. Samples were acidified with 10% v/v 0.01 M H_2SO_4. Measurement of acetate and propionate in cecal contents was performed using an Alliance separations module (e2695, Waters) with a 2489 UV/Vis detector and a C18 column (5 µm, 4.6 × 250 mm, XBridge, Waters). 0.01 M H_2SO_4 was used as the mobile phase. Acetate and propionate were identified by sample peak retention times that match the standards. Concentrations of acetate and propionate were determined by an external standard calibration method using Empower 3 (Waters).

Nuclear magnetic resonance measurement of targeted cecal metabolites

Cecal contents were collected immediately after mice were sacrificed and snap frozen for storage at -80°C. Before the experiment, samples were thawed and resuspended in 1 mL phosphate buffer (mix of 4 volumes of 1.5 M K_2HPO_4 with 1 volume of 1.5 M KH_2PO_4 and 1 mg/mL TSP in D_2O) per 500 mg cecal content sample. Centrifuge the homogenized sample at 17,000 × g for 15 min at 4°C and the supernatant was passed through a 0.22-µm syringe filter. The flow-through was transferred to an NMR tube for measurement and analysis. Bruker Avance III 600 NMR spectrometer was used to acquire a 1H-NMR spectrum. Data processing was performed using TopSpin 3.5 (Brucker) and data analysis was performed using Chenomx Profiler and Processor.

Isolation of mouse primary cardiac fibroblasts

To isolate cardiac fibroblasts from adult mice, ten-week-old C57BL/6J mice were anesthetized

and the chest cavity was exposed. The descending aorta was cut and the hearts were perfused with 1X phosphate buffered saline (PBS) (pH 7.4, Gibco) through the left ventricle before being harvested. Hearts were trimmed into small pieces and incubated in 0.025 mg/mL of Liberase (Roche) with 40 μg/mL DNase I (Roche) in Dulbecco's modified Eagle medium (DMEM), high glucose (Gibco) for 40 min at 37°C in a rotation incubator. The digested heart samples were filtered by a 70 μm cell strainer and the supernatants were filtered by a 40 μm cell strainer. The resulting supernatants were centrifuged at 450 × g for 5 min. The cells were then resuspended in DMEM with 10% fetal bovine serum and plated in 6-well plates. The medium was changed every day until the cells were expanded to 70% confluence.

Flow cytometric analysis of myofibroblast transformation

To assess the effect of acetate and propionate in inhibiting myofibroblast formation after TGF-β1 treatment, cardiac fibroblasts pretreated with acetate and/or propionate for 24 h followed by a 48-h treatment of TGF-β1 with acetate and/or propionate in combination were harvested, fixed, and stained with anti-vimentin (V2258, Sigma, 1:250) and anti-α-SMA (A2547, Sigma, 1:200) on ice for 30 min followed by fluorochrome-conjugated secondary antibodies on ice for 30 min. Flow cytometry was performed using a LSR II 17 color flow cytometer and the FACSDiva software (both products of BD Bioscience). Vimentin and α-SMA double-positive cells were quantified by the FlowJo software.

Western blotting

For detection of α-SMA, COL1A1, GPR41, GPR43, and GAPDH, cardiac fibroblasts pretreated with C2 and C3 for 24 h were treated with TGF-β1, C2, and C3 in combination for 48 h, and the cell lysates were collected by washing cells with cold PBS twice and lysing them in RIPA buffer containing 150 mM sodium chloride (Sigma), 50 mM Tris-HCl (Roche), 1% Nonidet P-40 (Boston bioproducts), 0.5% sodium deoxycholate (Sigma), and 0.1% SDS (Merck) with 1X protease and phosphatase Inhibitor cocktail (Halt, Life Technologies). For detection of total SMAD2, phosphorylated SMAD2, AMPK, phosphorylated AMPK, c-Jun, and phosphorylated c-Jun, lysates of cells treated with TGF-β1, C2, and C3 in combination for 4 h following 24-h-pretreatment of C2 and C3 were used. Lysates were centrifuged at 13,000 × g and 4°C for 20 min and the resulting supernatants were then collected for Western blot analysis. Protein concentrations were determined using Pierce BCA Protein Assay Kit (Thermo Fisher Scientific). Twenty micrograms of proteins were separated by 8% SDS polyacrylamide gel electrophoresis and transferred to polyvinylidene difluoride membranes (Immun-Blot, Bio-Rad Laboratories). The membranes were blocked with 5% bovine serum albumin (Sigma) in TRIS-buffered saline (TBS) at RT for 1 h and incubated with individual primary antibodies at 4°C overnight. The following antibodies were used: rabbit polyclonal anti-COL1A1 (GTX112731, GeneTex, 1:500), rabbit polyclonal anti-GPR41 (ab236654, Abcam, 1:1,000), rabbit polyclonal anti-GPR43 (bs-

13536R, Bioss Antibodies, 1:1,000), mouse monoclonal anti-GAPDH (MAB374, Merck, 1:2,000), rabbit polyclonal anti-α-SMA (ab5694, Abcam, 1:1,000), rabbit monoclonal anti-SMAD2 (5339, Cell Signaling, 1:1,000), rabbit polyclonal anti-SMAD2 (phospho S467) (ab280888, Abcam, 1:1,000), mouse monoclonal anti-AMPK (GTX42789, GeneTex, 1:1,000), rabbit monoclonal anti-phospho-AMPKα (Thr172) (2535, Cell Signaling, 1:1,000), rabbit monoclonal anti-c-Jun (9165, Cell Signaling, 1:1,000), and rabbit polyclonal anti-phospho-c-Jun (Ser63) (9261, Cell Signaling, 1:1,000) antibodies. After three washing steps with TBS with 0.05% Tween-20, membranes were incubated with secondary HRP-conjugated goat anti-mouse IgG (GTX213111-01, GeneTex, 1:2,000) or goat anti-rabbit IgG (7074, Cell Signaling, 1:2,000) antibodies at RT for 1 h. Visualization of bound antibodies was performed by the enhanced chemiluminescence (ECL) procedure as described by the manufacturer (Thermo Fisher Scientific). The optical density of each band was normalized to the corresponding GAPDH band. The blots were quantified using ImageJ software.

Collagen gel contraction assay for cardiac fibroblast contractility measurement

To assess the contractility of cardiac fibroblasts, 1.5×10^6 cells pretreated with acetate and/or propionate for 24 h followed by a 48-h treatment with TGF-β1 in combination with acetate and/or propionate were mixed with 2 mg/mL Collagen I (Gibco) and loaded into 24-well plates. The plate was incubated for 15 min at 37°C before adding DMEM with 10% fetal bovine serum and the gels were gently detached from the plate. The gels were incubated overnight and images of gels were captured for quantification of the gel area.

Knockdown of GPR41 and GPR43 in cardiac fibroblasts

The shRNAs targeting GPR41 (CCGGTCCTTCCTATCCTCTCATAAACTCGAGTTTATGAGAGGATAGGAAGGATTTTTG) and GPR43 (CCGGCCGGCCACTGTATGGAGTGATCTCGAGATCACTCCATACAGTGGCCGGTTTTT) were designed and constructed by the RNAi Core of Academia Sinica, Taiwan. The shRNAs were transfected into cardiac fibroblasts by transfection reagents (Lipofectamine 2000, Invitrogen) at a final concentration of 100 nM following the manufacturer’s instruction. The culture medium was changed 6 h after transfection and the knockdown efficiency was tested 48 h after transfection by Western blotting. Stable clones were selected and expanded for treatment of acetate, propionate, and TGF-β1 to examine the anti-fibrogenic effect of acetate and propionate.

Supplemental figures and table

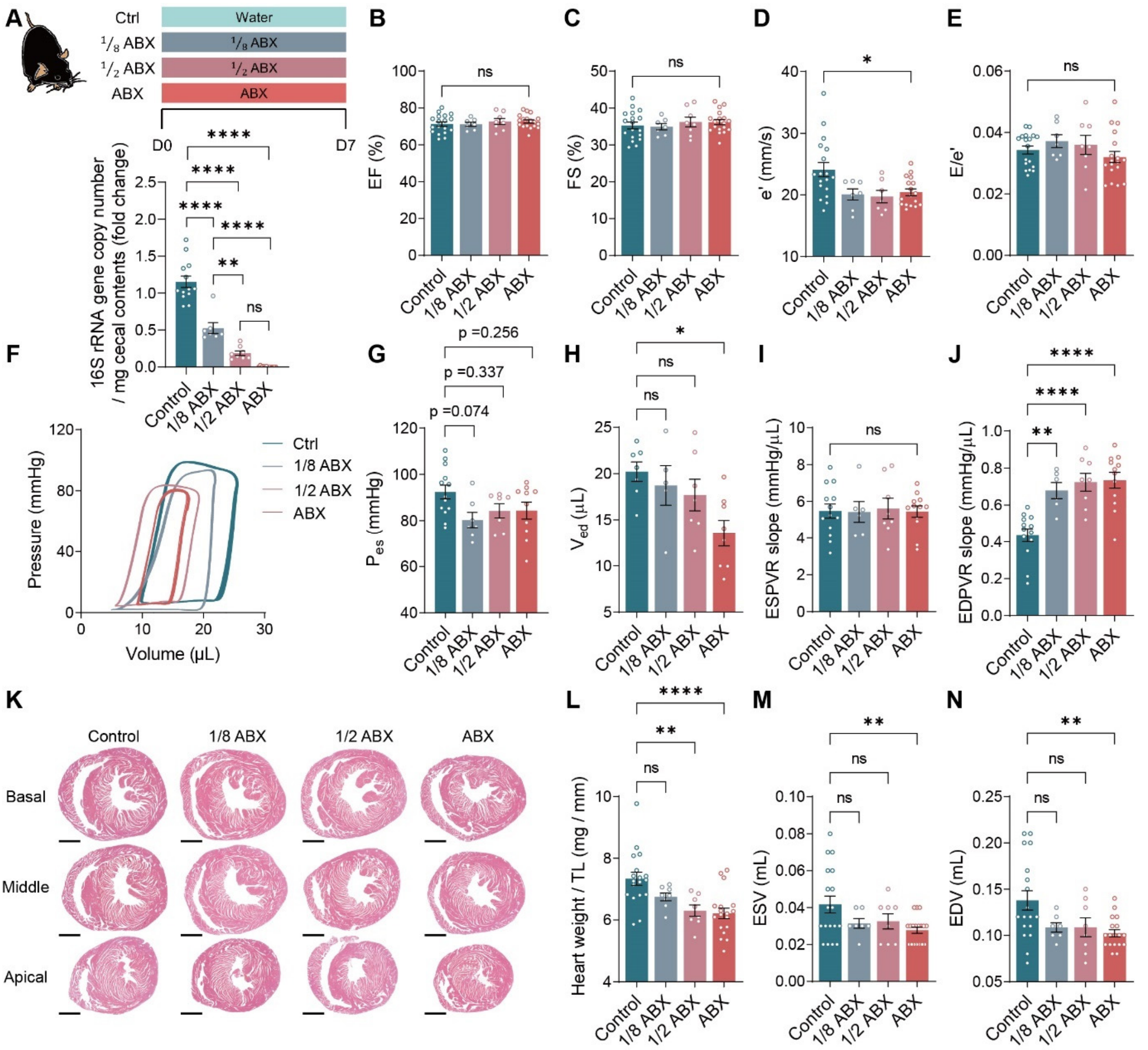

Figure S1. Depletion of gut microbiota induces heart morphological change and diastolic dysfunction.

A, Experimental design for testing the effect of gut microbiota depletion on mouse heart function and antibiotic treatment (ABX) efficiency. **B** through **E**, Heart systolic function was estimated by ejection fraction (EF) (**B**) and fractional shortening (FS) (**C**); diastolic function was estimated by the early diastolic mitral annular velocity (e') area (**D**) and the ratio of the peak early mitral inflow velocity over e' (E/e') (**E**) via echocardiography. **F** through **J**, Heart function was assessed by cardiac catheterization-derived pressure-volume loops (**F**); end-systolic pressure (Pes) (**G**), end-diastolic volume (Ved) (**H**), end-systolic pressure-volume relationship (ESPVR) slope representing cardiac contractility (**I**) whereas end-diastolic pressure-volume relationship (EDPVR) slope measuring myocardial passive stiffness is a diastolic function

indicator (**J**). **K**, HE stains of hearts of WT mice and mice depleted of gut microbiota. Scale bar, 1 mm. **L**, Cardiac hypertrophy was estimated by heart weight normalized by tibial length (TL). **M** and **N**, Left ventricular dilation was measured by left ventricular internal diameter at end-systole (LVIDs) (**M**) and LVID at end-diastole (LVIDd) (**N**). Statistical significance was determined by ordinary one-way ANOVA with Tukey multiple comparisons test. ns, not significant; *, $P < 0.05$; **, $P < 0.01$; ****, $P < 0.0001$.

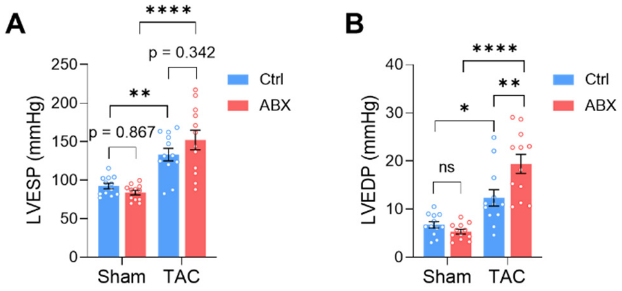

Figure S2. Depletion of gut microbiota exacerbates resting left ventricular pressure elevation after pressure overload.

A and B, Left ventricular pressure at end-systole (LVESP) (A) and left ventricular pressure at end-diastole (LVEDP) derived from cardiac catheterization. Statistical significance was determined by two-way ANOVA with Tukey multiple comparisons test. ns, not significant; *, $P < 0.05$; **, $P < 0.01$; ****, $P < 0.0001$.

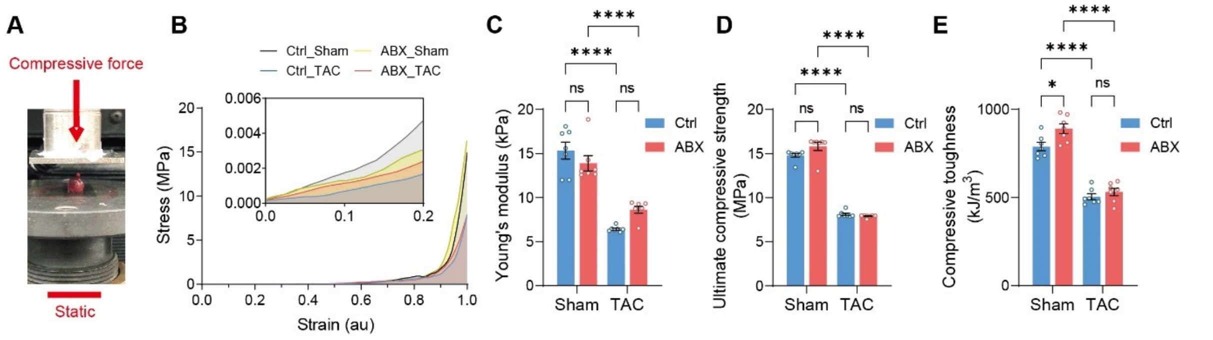

Figure S3. Gut microbiota depletion changes heart compressive strength.

A, A representative image of the heart sample set for the compressive test. **B**, Representative stress-strain curves of hearts under compression. **C** through **E**, Compressive test-derived mechanical properties including Young's modulus (**C**), Ultimate strength (**D**), and toughness (**E**) of mouse hearts. Statistical significance was determined by two-way ANOVA with Tukey multiple comparisons test. ns, not significant; *, $P < 0.05$; ****, $P < 0.0001$.

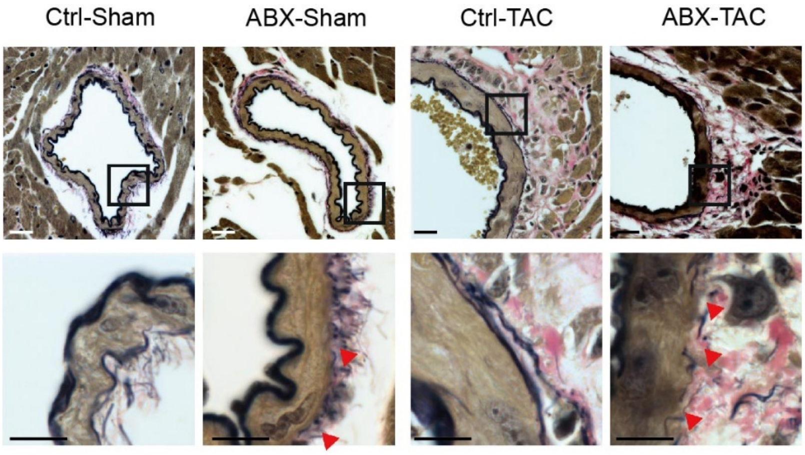

Figure S4. Elastic fiber fragmentation in gut microbiota-depleted mouse hearts. Representative Verhoeff-Van Gieson-stained heart sections showing elastin fibers in black and collagen fibers in red. Upper scale bar, 20 µm; Lower scale bar, 10 µm.

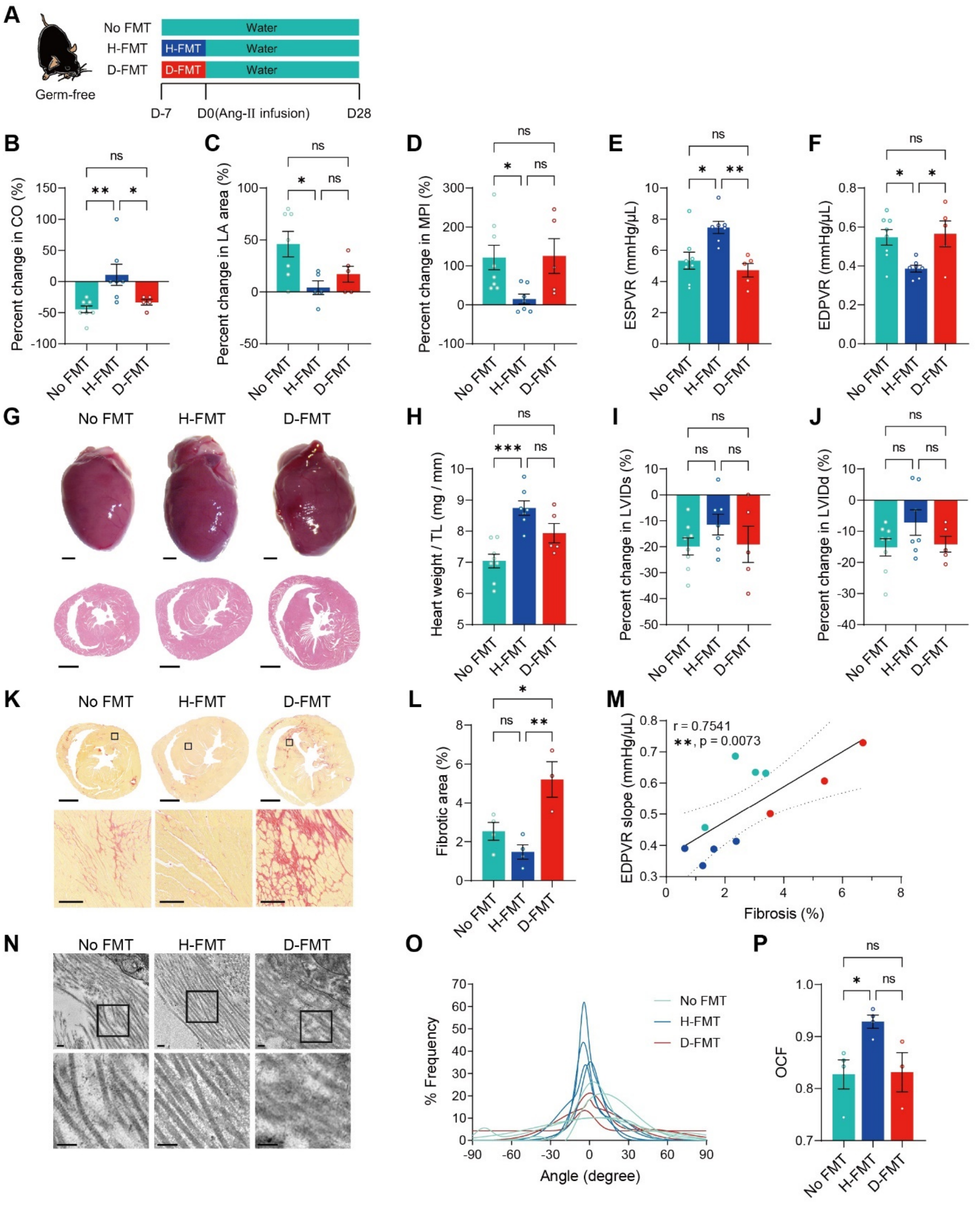

Figure S5. Healthy gut microbiome benefits heart function and ECM remodeling in germ-free mice under hypertrophic stress.

A, Experimental design for testing the effect of gut microbiota reconstruction on germ-free mice after Ang-II infusion. **B** and **C**, Changes of heart systolic function indicated by cardiac output (CO) (**B**) and diastolic function indicated by left atrial (LA) area (**C**) one week before and four weeks after Ang-II infusion were assessed by echocardiography. LA area of one no FMT

and two H-FMT mice were unattainable due to limitation of image quality. **D**, Changes in global heart function indicated by myocardial performance index (MPI) were assessed by pulsed-wave Doppler. **E** and **F**, Cardiac catheterization-derived pressure-volume relationship at end-systole (ESPVR) (**E**) and end-diastole (EDPVR) (**F**) measuring cardiac contractility and myocardial passive stiffness, respectively. **G**, Representative bright-field images of whole hearts and hematoxylin and eosin stained heart sections. Scale bar, 1 mm. **H**, Cardiac hypertrophy was assessed by heart weight normalized by tibial length (TL). **I** and **J**, Changes in left ventricular internal dimensions at end-systole (LVIDs) and end-diastole (LVIDd) one-week before and four-week after Ang-II infusion. **K** and **L**, Representative images of picrosirius red-stained heart sections; upper scale bar, 1 mm; lower scale bar, 100 µm (**K**) and quantification of cardiac fibrotic area (**L**). **M**, Relation between EDPVR slope and fibrosis determined by simple linear regression. The solid line indicates the best-fit line, the dashed lines above and below it indicates the upper and lower limit of the 95% confidence intervals of the linear regressions, respectively, and r is the correlation coefficient. **N**, Collagen fibrils under the transmission electron microscope. Scale bar, 0.2 µm. **O**, Collagen fibril angular distribution. **P**, Orientation correlation function (OCF) for quantification of fiber alignment. Statistical significance was determined by ordinary one-way ANOVA with Tukey multiple comparisons test in **B** through **F**, **H**, **I**, **J**, **L**, and **P**. ns, not significant; *, $P < 0.05$; **, $P < 0.01$; ***, $P < 0.001$; ****, $P < 0.0001$.

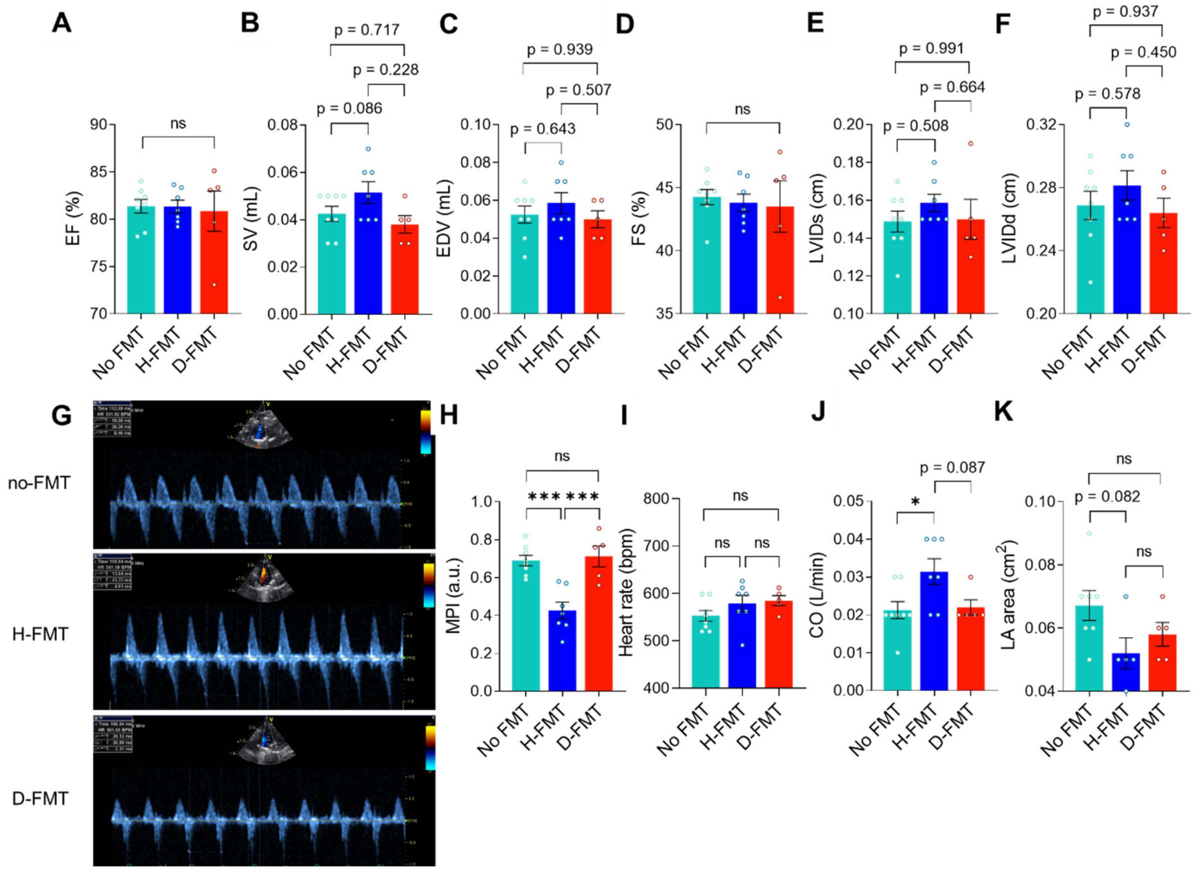

Figure S6. Echocardiography-derived cardiac functional assessment in germ-free mice with or without cecal microbiota transplantation.

A through **F**, Heart function and ventricular volume parameters including ejection fraction (EF) (**A**), stroke volume (SV) (**B**), end-diastolic volume (EDV) (**C**), fractional shortening (FS) (**D**), and left ventricular internal dimension at end-systole (LVIDs) (**E**) and end-diastole (LVIDd) (**F**) were assessed by M-mode echocardiography 28 days after Ang-II. **G** and **H**, Representative Doppler echocardiographic images (**G**) for calculation of myocardial performance index (MPI) by summation of isovolumic contraction time (ICRT) and isovolumic relaxation time (IVRT) divided by ejection time (ET) (**H**). **I** through **K**, Heart rate (**I**), cardiac output (**J**), and left atrial (LA) area (**K**) of mice 28 days after Ang-II. LA area of one no FMT and two H-FMT mice were unattainable due to limitation of image quality. Statistical significance was determined by ordinary one-way ANOVA with Tukey multiple comparisons test. ns, not significant; *, $P < 0.05$; **, $P < 0.01$; ****, $P < 0.0001$.

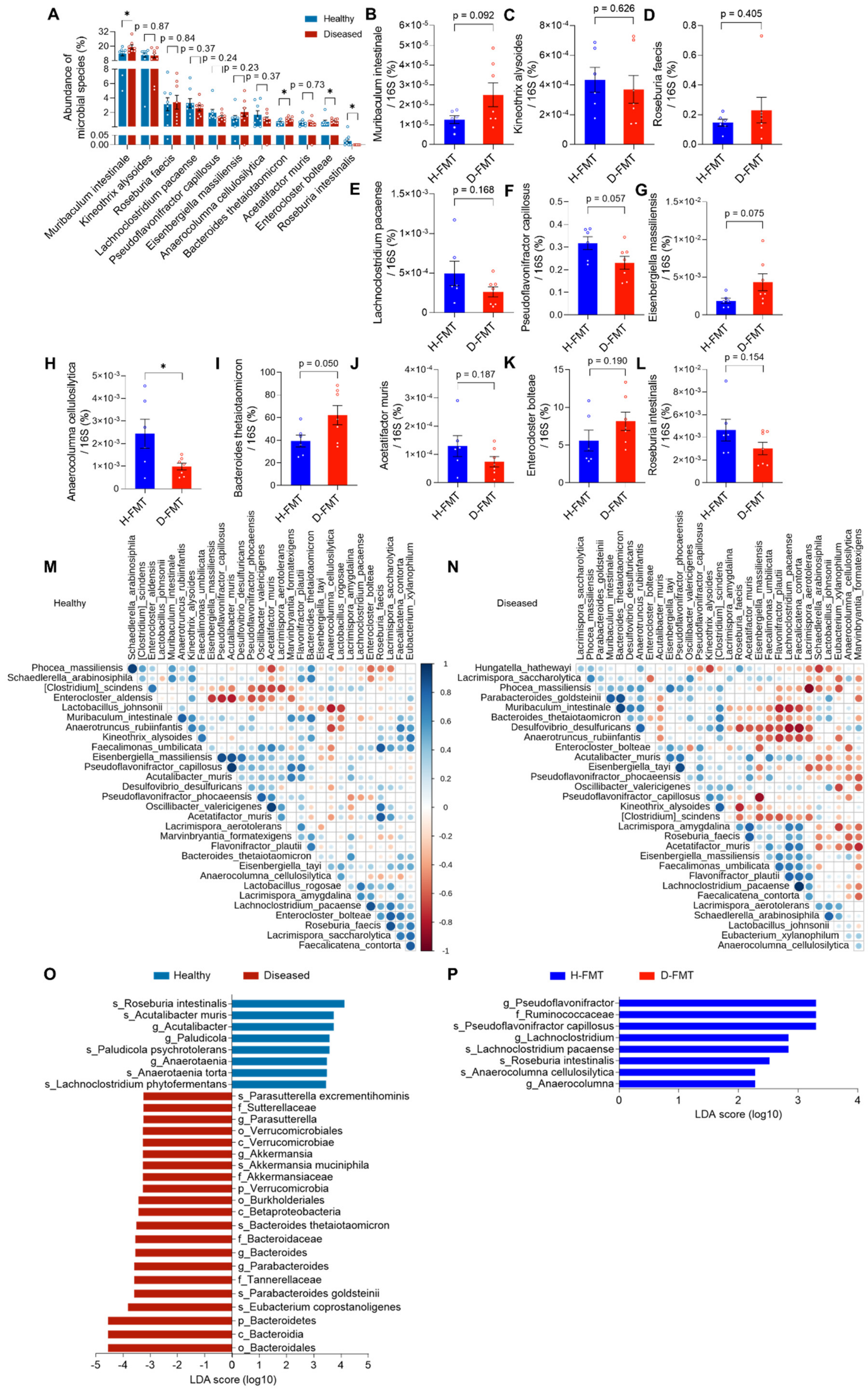

Figure S7. The difference in gut microbial composition between healthy and diseased donors was re-established in SPF microbiota transplantation recipients.

A, The abundance of gut microbes found to be dominant in healthy or diseased donors were quantified by 16S rRNA sequencing. **B** through **L**, Abundance of Muribaculum intestinale (**B**), Kineothrix alysoides (**C**), Roseburia faecis (**D**), Lachnoclostridium pacaense (**E**), Pseudoflavonifractor capillosus (**F**), Eisenbergiella massiliensis (**G**), Anaerocolumna cellulosilytica (**H**), Bacteroides thetaiotaomicron (**I**), Acetatifactor muris (**J**), Enterocloster bolteae (**K**), and Roseburia intestinalis (**L**) in SPF gut microbiota transplantation recipients was determined by qPCR with primers specific to each bacterial species. **M** and **N**, Spearman correlation analysis of gut microbiome of healthy donors (**M**) and diseased donors (**N**). **O** and **P**, Linear discriminant analysis (LDA) score of gut microbial taxa in healthy and diseased microbiota transplantation donors (**O**) and donors (**P**). Statistical significance was determined by a multiple unpaired t-test with FDR set at Q = 1% in **A.** Statistical significance was determined by an unpaired two-tailed t-test in **B** through **L**. *, $P < 0.05$.

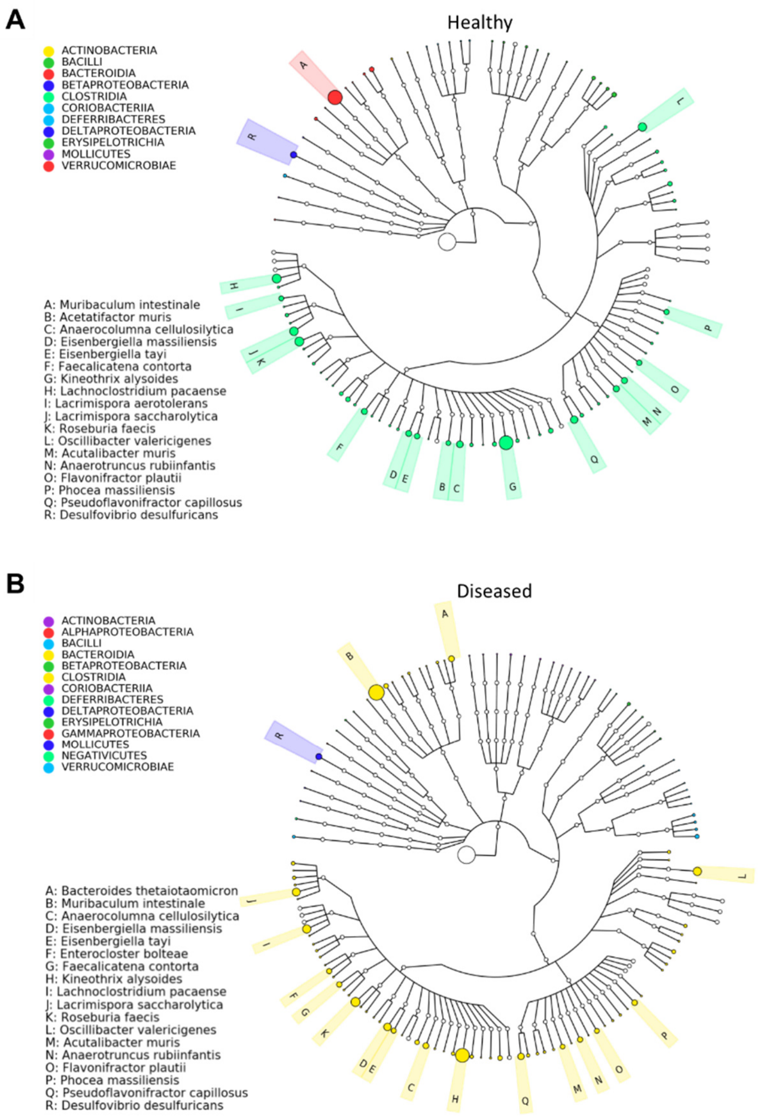

Figure S8. Gut microbiota composition in healthy and diseased donors.

A and **B**, Gut bacteria phylogenetic tree derived from graphical phylogenetic analysis of healthy donors (**A**) and diseased donors (**B**).

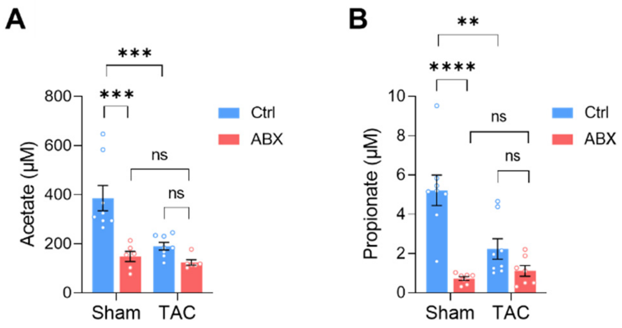

Figure S9. Circulating acetate and propionate levels in microbiota transplantation donor mice.

Quantification of acetate (A) and propionate (B) in the cecal contents of donors for microbiota transplantation experiments as well as antibiotics-treated mice. Statistical significance was determined by two-way ANOVA with Tukey multiple comparisons test. ns, not significant; **, $P < 0.01$; ***, $P < 0.001$; ****, $P < 0.0001$.

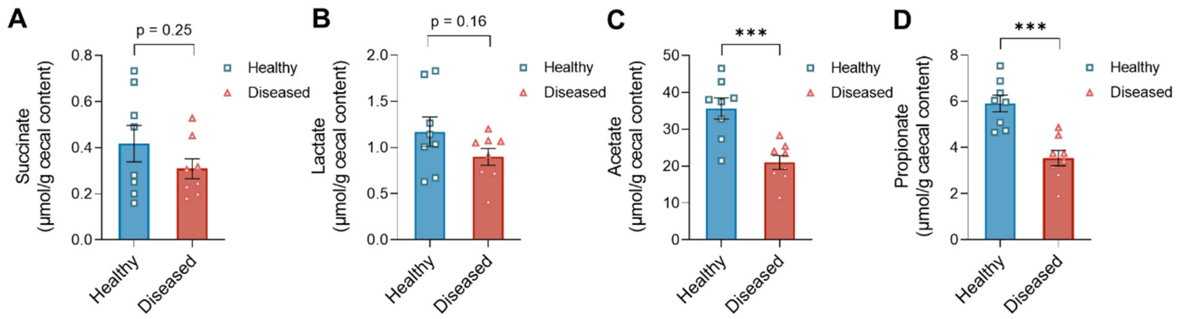

Figure S10. Cecal level of metabolites involved in acid fermentation and acetylene degradation pathways enriched by the gut microbiota of healthy donors determined by nuclear magnetic resonance spectrometry.

Quantification of succinate (**A**), lactate (**B**), acetate (**C**), and propionate (**D**) in the cecal contents of donors for microbiota transplantation experiments. Statistical significance was determined by an unpaired two-tailed t-test. ***, $P < 0.001$.

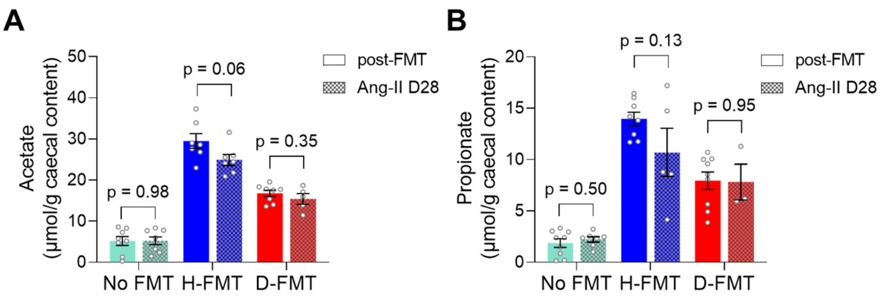

Figure S11. Ang-II affects fecal acetate and propionate levels in mice with healthy cecal microbiota transplantation.

A and **B**, Fecal acetate (**A**) and propionate (**B**) levels before and after Ang-II. Statistical significance was determined by a multiple unpaired t-test with FDR set at Q = 1%.

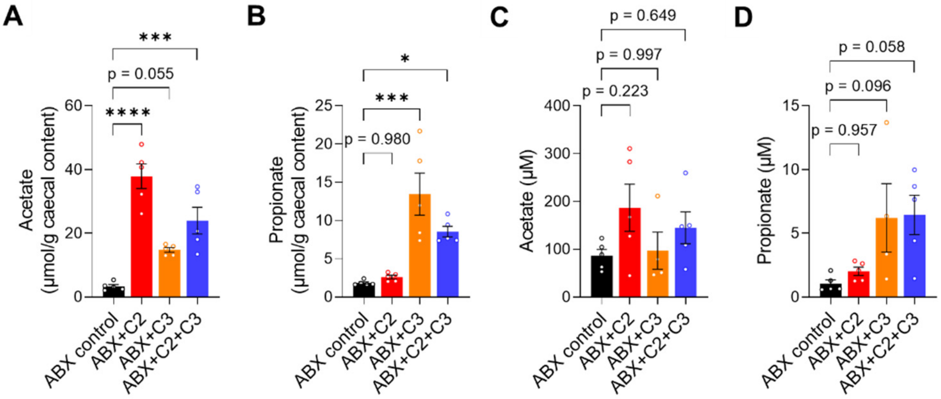

Figure S12. Cecal and circulating acetate and propionate levels on TAC D28 in acetate- and propionate-supplemented mice.

A and **B**, Quantification of cecal level of acetate (**A**) and propionate (**B**) in mice supplemented with acetate (C2), propionate (C3), or both. **C** and **D**, Quantification of acetate (**C**) and propionate (**D**) in the plasma of mice supplemented with C2. C3, or combined. Statistical significance was determined by ordinary one-way ANOVA with Tukey multiple comparisons test. ns, not significant; *, $P < 0.05$; ***, $P < 0.001$; ****, $P < 0.0001$.

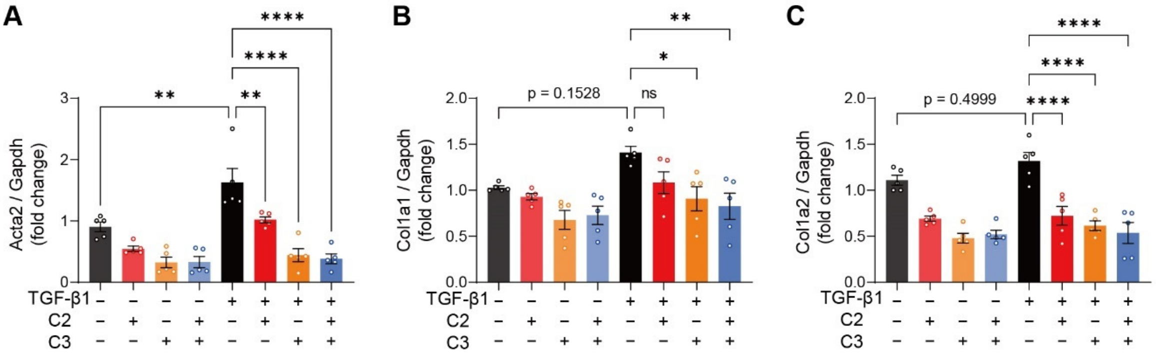

Figure S13. ECM gene expression is suppressed by acetate and propionate in TGF-β1-treated cardiac fibroblasts.

A through **C**, Gene expression of Acta2 (**A**), Col1a1 (**B**), and Col1a2 (**C**) in acetate- and propionate-treated cardiac fibroblasts. Statistical significance was determined by ordinary one-way ANOVA with Tukey multiple comparisons test. ns, not significant; *, *P* < 0.05; **, *P* < 0.01; ****, *P* < 0.0001.

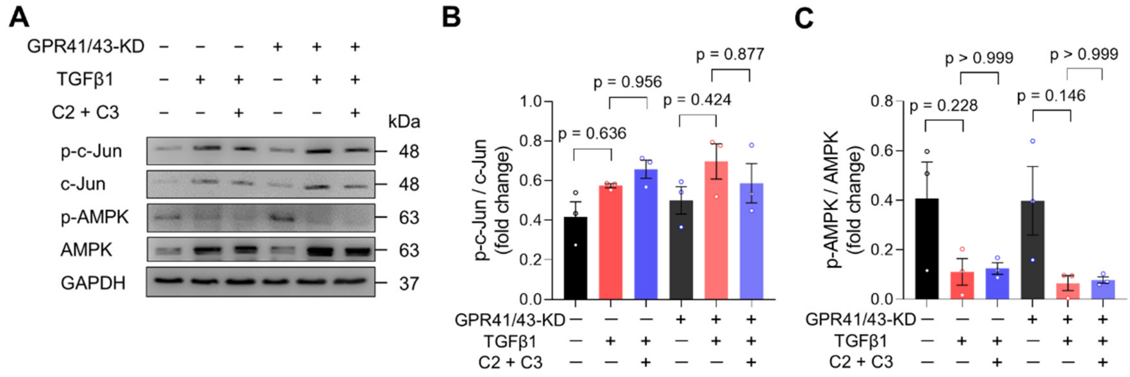

Figure S14. Acetate and propionate inhibit TGF-β1-induced cardiac fibroblast activation independent of AMPK/c-Jun signaling pathway.

A through **C**, Western blotting of c-Jun and AMPK protein expression in GPR41 and GPR43 double knockdown cardiac fibroblasts (**A**) with quantification of phosphorylated c-Jun (**B**) and phosphorylated AMPK (**C**) protein expression. Statistical significance was determined by ordinary one-way ANOVA with Tukey multiple comparisons test.

Table S1. Products of predicted pathways enriched in healthy and diseased donors.

Pathway	Enriched group	Product
Tetrapyrrole biosynthesis from glycine	Healthy	uroporphyrinogen-III, ammonium, CO_2, coenzyme A
Mixed acid fermentation	Healthy	NAD^+, 2-oxoglutarate, CO_2, NADPH, a menaquinone, succinate, H_2, *(R)* -lactate, ATP, ethanol, acetate
Acetylene degradation (anaerobic)	Healthy	ethanol, ATP, acetate
Guanosine ribonucleotides *de novo* biosynthesis	Healthy	GTP, ADP, H^+, L-glutamate, diphosphate, AMP, NADH
Poly(glycerol phosphate) wall teichoic acid biosynthesis	Healthy	AMP, diphosphate, ADP, phosphate, UMP, CMP, *di-trans,octa-cis* -undecaprenyl diphosphate, UDP
Peptidoglycan biosynthesis II (staphylococci)	Healthy	L-glutamate, *ditrans,octacis* -undecaprenyldiphospho-*N* -acetyl-(*N* -acetylglucosaminyl)muramoyl-L-alanyl-γ-D-isoglutaminyl-L-lysyl-D-alanyl-D-alanine, ADP, phosphate, H^+, *di-trans,octa-cis* -undecaprenyl diphosphate, D-alanine, UDP, UMP
TCA cycle IV (2-oxoglutarate decarboxylase)	Diseased	an electron-transfer quinol, NADH, CO_2, NADPH, coenzyme A
Pyridoxal 5'-phosphate biosynthesis I	Diseased	CO_2, NADH, 2-oxoglutarate, phosphate, hydrogen peroxide, pyridoxal 5'-phosphate
Fatty acid biosynthesis initiation	Diseased	coenzyme A, an acetoacetyl-[acp], CO_2
Chondroitin sulfate degradation I	Diseased	4-deoxy-L-*threo* -hex-4-enopyranuronate, *N* -acetyl-D-galactosamine, *N* -acetyl-D-galactosamine 6-O-sulfate, H^+, sulfate
L-rhamnose degradation I	Diseased	H^+, ADP, (*S*)-lactaldehyde, glycerone phosphate
Pyridoxal 5'-phosphate biosynthesis and salvage	Diseased	pyridoxal 5'-phosphate
(5Z)-dodecenoate biosynthesis I	Diseased	H_2O, CO_2, $NADP^+$, NAD^+
Palmitoleate biosynthesis I	Diseased	palmitoleate, CO_2, $NADP^+$, NAD^+
Stearate biosynthesis II	Diseased	stearoyl-CoA, AMP, diphosphate, NAD^+, $NADP^+$, CO_2
Oleate biosynthesis IV (anaerobic)	Diseased	H_2O, NAD^+, $NADP^+$, CO_2
Urea cycle	Diseased	ADP, phosphate, H^+, urea, fumarate, diphosphate, AMP
Mycolate biosynthesis	Diseased	$NADP^+$, CO_2, NAD^+, *S* -adenosyl-L-homocysteine, arabinogalactan-trans-keto-mycolate, α,α-trehalose, arabinogalactan-cis-keto-mycolate, arabinogalactan-trans-methoxy-mycolate, arabinogalactan-cis-methoxy-mycolate, trehalose-trans-keto-di-mycolate, trehalose-cis-keto-di-mycolate, trehalose-trans-methoxy-di-mycolate, trehalose-cis-methoxy-di-mycolate, α,α'-trehalose 6,6'-bismycolate, ADP, phosphate, AMP, diphosphate
Pyrimidine deoxyribonucleotides de novo biosynthesis	Diseased	pyrimidine deoxyribonucleotides
Palmitate biosynthesis	Diseased	lauroyl-CoA, diphosphate, AMP, palmitoyl-CoA, NAD^+, $NADP^+$, CO_2
8-amino-7-oxononanoate biosynthesis I	Diseased	(8*S*)-8-amino-7-oxononanoate, CO_2, *S* -adenosyl-L-homocysteine, $NADP^+$, NAD^+, methanol
Histidine, purine, and pyrimidine biosynthesis	Diseased	histidine, purine, pyrimidine
Biotin biosynthesis I	Diseased	biotin
Tetrahydrofolate biosynthesis	Diseased	tetrahydrofolate
4-deoxy-L-*threo* -hex-4-enopyranuronate degradation	Diseased	D-glyceraldehyde 3-phosphate, pyruvate, ADP, NAD^+
Tetrahydrofolate biosynthesis and salvage	Diseased	tetrahydrofolate
